# Key Maize Drought-Responsive Genes and Pathways Revealed by Comparative Transcriptome and Physiological Analyses of Contrasting Inbred Lines

**DOI:** 10.3390/ijms20061268

**Published:** 2019-03-13

**Authors:** Tinashe Zenda, Songtao Liu, Xuan Wang, Guo Liu, Hongyu Jin, Anyi Dong, Yatong Yang, Huijun Duan

**Affiliations:** 1 Department of Crop Genetics and Breeding, College of Agronomy, Hebei Agricultural University, Baoding 071001, China; tzenda@hebau.edu.cn (T.Z.); m15028293845@163.com (S.L.); 15733289921@163.com (X.W.); m15612245597@163.com (G.L.); m15633790536@163.com (H.J.); 18331220513@163.com (A.D.); 18233230155@163.com (Y.Y.); 2 North China Key Laboratory for Crop Germplasm Resources of the Education Ministry, Hebei Agricultural University, Baoding 071001, China

**Keywords:** differentially expressed genes (DEGs), drought stress, qRT-PCR, RNA sequencing (RNA-seq), transcriptome, *Zea mays* L.

## Abstract

To unravel the molecular mechanisms underpinning maize (*Zea mays* L.) drought stress tolerance, we conducted comprehensive comparative transcriptome and physiological analyses of drought-tolerant YE8112 and drought-sensitive MO17 inbred line seedlings that had been exposed to drought treatment for seven days. Resultantly, YE8112 seedlings maintained comparatively higher leaf relative water and proline contents, greatly increased peroxidase activity, but decreased malondialdehyde content, than MO17 seedlings. Using an RNA sequencing (RNA-seq)-based approach, we identified a total of 10,612 differentially expressed genes (DEGs). From these, we mined out four critical sets of drought responsive DEGs, including 80 specific to YE8112, 5140 shared between the two lines after drought treatment (SD_TD), five DEGs of YE8112 also regulated in SD_TD, and four overlapping DEGs between the two lines. Drought-stressed YE8112 DEGs were primarily associated with nitrogen metabolism and amino-acid biosynthesis pathways, whereas MO17 DEGs were enriched in the ribosome pathway. Additionally, our physiological analyses results were consistent with the predicted RNA-seq-based findings. Furthermore, quantitative real-time polymerase chain reaction (qRT-PCR) analysis and the RNA-seq results of twenty representative DEGs were highly correlated (*R*^2^ = 98.86%). Crucially, tolerant line YE8112 drought-responsive genes were predominantly implicated in stress signal transduction; cellular redox homeostasis maintenance; *MYB*, *NAC*, *WRKY*, and *PLATZ* transcriptional factor modulated; carbohydrate synthesis and cell-wall remodeling; amino acid biosynthesis; and protein ubiquitination processes. Our findings offer insights into the molecular networks mediating maize drought stress tolerance.

## 1. Introduction

Drought remains the primary abiotic constraint to plant growth and development, as well as crop productivity [1,2]), accounting for approximately 70% potential yield loss worldwide, largely owing to climate change [3,4]. More startling, current global climate change models predict more frequent and severe extreme weather events, along with the general temperature increase [5,6]. Consequently, water deficiency is expected to become worse and its impact on the physiological status and productivity of agronomically important plants is expected to become even more relevant during the next few decades [7]. 

Worldwide, maize (*Zea mays* L.) is the third most essential food crop after wheat (*Triticum aestivum* L.) and rice (*Oryza sativa* L.) [8]. However, like most of the cereal crops on which the world population depends for food, maize productivity is threatened by drought. Whereas the maize crop is more sensitive to drought at pre-anthesis and grain-filling periods [9], drought stress at the seedling stage can also be devastating [3]. Yield loss emanating from seedling-stage-drought-stress is of major concern in arid and semi-arid areas, such as Hebei Province in Northern China, where maize often experience moisture deficit stress in spring and early summer, thereby threatening germination and seedling growth [10]. Generally, compared to late vegetative and anthesis growth phases, the maize seedling phase has less water demands [11]. However, moisture deficit at the seedling phase will hamper early crop establishment and negatively impact on plants’ grain yield potential, as a consequence of premature tasseling and a prolonged anthesis-silk interval [12]. Therefore, untying the molecular basis of maize seedling-stage drought response, in order to improve early crop establishment in such arid and semi-arid drought-prone regions, remains pertinent in maize breeding programs [3].

To cope with drought stress, plants have evolved complex adaptive mechanisms, including physiological and metabolic reprogramming, regulation of transcription and gene expression, as well as epigenetic plasticity [2,9,13]. Various genes are expressed and translated in response to water deficit conditions [14]. Several studies performed to understand the molecular mechanisms of drought stress response have identified conserved and species-specific drought responsive genes, including membrane stabilizing proteins and late embryogenic abundant proteins (LEA), which increases cells’ water binding capacity [15,16,17]. Several heat shock proteins (HSPs), which play a major role in stabilizing protein structure, were also identified [18,19,20]. The HSPs are chiefly involved in unwinding some folded proteins and averting protein denaturation under abiotic stress conditions [14]. Additionally, several transcription factors that also regulate and provide adaptive response under drought stress were identified, including myeloblastosis (MYB), dehydration responsive element binding (DREB), C-repeat binding factor (CBF), abscisic acid responsive elements binding factor (ABF), ABRE binding (AREB), (**NAM**, **ATAF**_1_/_2,_ and **CUC**_2_ containing proteins) (NAC), WRKY, and SNF1-related kinase 2 (SnRK2) [21,22,23]. Despite these achievements being made; however, the gene network of the drought stress response is still not fully elucidated [3]. Moreover, the existence of a variety of drought-inducible genes suggests that the nature of the response to drought stress is complex [9]. Therefore, elucidating drought-tolerance mechanisms will greatly enhance the development of new crop cultivars that are better adapted to regions most experiencing climate-change-exacerbated droughts, consequently leading to improved sustainable crop productivity and food security. 

Considerable scientific progress has been recorded in deciphering maize vegetative- and reproductive-stage drought response mechanisms [2]. Moreover, previous researchers [9,24,25] have attempted to untie the complex gene regulatory networks involved in maize inbred lines’ drought stress response at the seedling stage. Despite the recorded achievements in this respect, our understanding of the molecular mechanisms mediating maize seedling-stage drought response remains inadequate [3]. However, recent advances in molecular biology methods have offered us convenience in deciphering plants’ underlying response mechanisms to various abiotic stresses [8]. With the development of next generation sequencing, RNA sequencing (RNA-seq) has aided our convenience in carrying out transcriptome analyses of plants’ drought stress responses [2,26]. Using RNA-seq, we can obtain transcripts from the RNA in a tissue- or cell-specific manner, transcribed at a developmental stage or functional state, and this is fundamental to fishing out functional genes [27]. Furthermore, compared to traditional sequencing methods, RNA-seq boasts of its low cost, high-throughput, and high sensitivity [22,28]. Resultantly, our knowledge pertaining to gene expression networks modulating abiotic stress adaptation in plants has been improved [13]. Several studies [9,26,29,30,31] have employed the use of RNA-seq technology, and numerous genes that respond to abiotic stresses in various crop species have been identified. It is conceivable that the study of gene expression profiles and biological functions will enhance our in-depth comprehension of plants’ response to environmental stresses, thereby aiding in breeding better adapted crop species. 

Herein; therefore, using an illumina RNA sequencing and analysis-based method; we have performed a comprehensive comparative transcriptome analysis of two contrasting maize inbred lines (drought-tolerant YE8112 and drought-sensitive MO17) at the seedling (three-fully-expanded-leaves) stage, in order to elucidate the dynamic molecular mechanisms underpinning the drought stress responses in maize. Further, we have evaluated some physiological indices in order to have a systems biology approach to understanding plants’ drought response mechanisms. Our findings provide further elucidation of maize drought stress tolerance, in addition to providing a basis for further targeted cloning studies.

## 2. Results

### 2.1. Physiological Analysis of Two Contrasting Maize Inbred Line Seedlings Responses to Drought Stress

First, we exposed three-leaf-stage maize seedlings (for both tolerant line YE8112 and sensitive line MO17) to water-sufficient (control) and water-deficit (drought) treatment for seven days under greenhouse environment. Some physiological and phenotypic responses of the inbred lines to drought stress were then analyzed. In both maize inbred lines, leaf relative water content significantly (*p* ≤ 0.05) decreased with the increasing number of days of exposure to drought stress (Table 1). The decline in leaf relative water content in the sensitive line MO17 was evidently greater than that in the tolerant line YE8112 under drought stress conditions. Proline content and peroxidase (POD) enzyme activity significantly increased with increasing duration of stress exposure (Table 1). Comparatively, tolerant line YE8112 maintained relatively higher values of proline and POD contents than sensitive line MO17 at any given time point (Table 1). Results of the malondialdehyde (MDA) content showed that the parameter increased with the increasing number of stress exposure days, starting from day one in sensitive line MO17 and day three in tolerant line in YE8112. The significant increase in MDA content was greatest from day three to day five, becoming minimal thereafter, with sensitive line MO17 exhibiting comparatively higher values than tolerant line YE8112 (Table 1). This observation may indicate that, with the increase of drought exposure duration, leaf cell membranes are severely injured, consequently resulting in cell membrane lipid release and membrane structures destruction [32]. As we had already observed in our most recent study [32], phenotypically, the two inbred lines showed no apparent differences under control conditions. However, after seven days of drought treatment exposure, they exhibited some considerable variations, with tolerant line YE8112 maintaining fully expanded green leaves and intact plant stature, whilst sensitive line MO17 leaves became shriveled up and the plant structure became distorted (Table 1; [32]).

### 2.2. RNA Sequencing (RNA-seq) Analysis 

RNA for use in RNA-seq transcriptome analysis was isolated from the leaves of the three-leaf-stage maize seedlings that had been subjected to the drought treatment, as described in Section 2.1 above. Six samples for tolerant line YE8112 (T01, T02, T03, T04, T05, and T06) and six for the sensitive line MO17 (T07, T08, T09, T10, T11, and T12) were categorized into four groups, viz; (T01, T02, and T03), (T07, T08, and T09)—under water-sufficient conditions; and (T04, T05, and T06), (T10, T11, and T12)—under water-deficit conditions. These four groups were named as tolerant control (TC), sensitive control (SC), tolerant drought (TD), and sensitive drought (SD), respectively. We then analyzed the transcriptomes of the two lines in response to drought stress. The cDNA libraries were prepared from these two inbred lines, before (control, C) and after drought treatment (D), and then subjected to RNA-seq profiling on the Illumina Hiseq Xten platform (San Diego, CA, USA). The raw data were deposited in the NCBI Sequence Read Archive (SRA), under accession numbers SRR5989965–SRR5989974.

After filtering, a total of 98.61 GB of clean data was generated from ten samples (two unfit samples, T07 and T12, were retrieved). For the ten samples, we obtained 567.03 million total reads, with 56.70 million reads on average from each sample. The clean reads were mapped to the maize reference genome B73. The Q30 base percentage, an indicator of the overall reproducibility and quality of the assay, was above 85.0%, which met the demands for further analysis. The mapping rates ranged from 56.83% to 78.49% (Table 2). Interestingly, the percentage of mapped reads was lower in the samples from drought-stressed MO17 than in the other samples (Table 2). This might be because drought stress increases variation in transcripts in MO17. 

Mortazavi’s [33] method of fragments per kilobases of exon model per million mapped reads (FPKM) was used to measure the transcript abundance of each gene. To test the repeatability and reliability of the results, we tested the relation of expression patterns among the drought treatment (DT) and drought control (DC) replicates by Pearson’s correlation test (Figure S1). The RNA-seq correlation coefficients of FPKM, among DT and DC replicates, showed that the gene expression patterns were similar (Figure S1), thus confirming the repeatability and reliability of evaluation results. Further, to analyze the similarities and differences between the twelve samples, principal component analysis (PCA) of all samples was performed [33]. The PCA analysis showed that there was low consistence amongst the three replications of the two (one drought-stress and one control) samples, probably due to a technical failure (Figure S2). Thus, sequencing results were analyzed by retrieving samples T07 (MO17 water-sufficient) and T12 (MO17 water-deficit). The PCA results of the remaining 10 samples showed clear separation between the drought tolerant and drought sensitive lines. Additionally, the replicates of each treatment clustered together (Figure S2). These results showed that this experiment was reproducible and reliable.

### 2.3. Transcriptomic Responses

**Figure 1:**
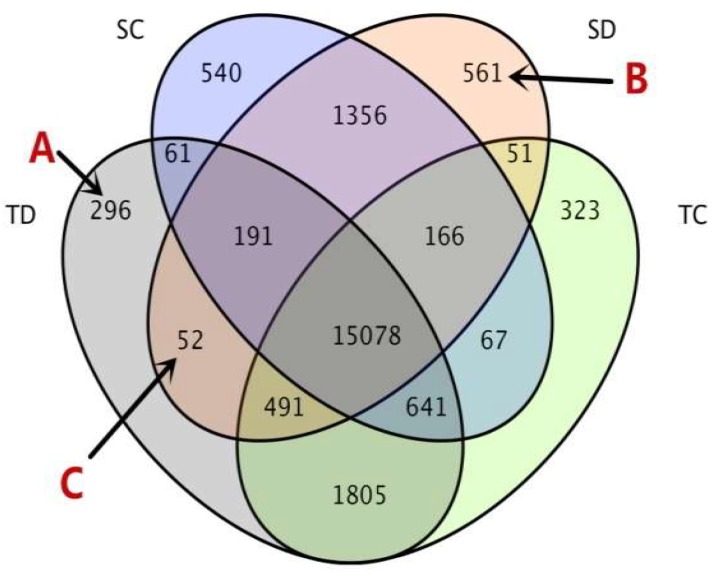
Profile of gene expression by inbred line and drought treatment. The gene expression profile is illustrated as the number of transcriptomic responses by using a Venn diagram. A total of 21,679 genes were expressed. Drought treatments are labeled Control (C) and Drought (D). The tolerant line (YE8112) and the sensitive line (MO17) are labeled “T” and “S”, respectively. The biological samples of four combinations are TC, TD, SC, and SD, respectively. The area labeled “A” represents the genes exclusively expressed in tolerant line YE8112 after drought treatment (TD). The area labeled “B” represents the genes specifically expressed in sensitive line MO17 after drought treatment (SD). The area labeled “C” represents the drought responsive genes shared by the tolerant and sensitive lines (i.e., TD and SD, but excluding SC and TC).

In the current study, fragments per kilobase of exon model per million mapped reads (FPKM) values ≥ 1 were used to determine genes expressed. Using Cufflinks software package [34], a total of 21,679 annotated gene transcripts was identified from the four (TC, SC, TD, SD) drought treatments. The Venn diagram (Figure 1) shows the number of genes exclusively expressed in each treatment, overlapping genes between treatments, and common genes among all treatment combinations. Of these 21,679 gene transcripts, 69.55% (15,078) were represented in all treatments. Before drought stress, 83.49% (18,100) and 85.90% (18,622) of the genes were expressed in the sensitive line (MO17, SC) and the tolerant line (YE8112, TC), respectively. After drought stress, 82.78% (17,946) and 85.87% (18,615) were expressed in MO17 (SD) and YE8112 (TD), respectively (Figure 1). 

A total of 296 genes (Group A in Figure 1) were exclusively expressed in tolerant line YE8112 after drought treatment (TD). Gene ontology (GO) annotation analysis (agriGO, http://systemsbiology.cau.edu.cn/agriGOv2/#, accessed on 13 October 2018) of this group showed that GO: 0008152 (metabolic process), GO: 0009987 (cellular process), and GO: 0050896 (response to stimulus) were the most significantly enriched GO terms in the biological process (BP) category. Within the molecular function (MF) category, GO: 0005488 (binding) and GO: 0003824 (catalytic activity) were most significantly enriched (Table S1, Sheet 1).

Group B, containing 561 genes, represents the genes specifically expressed in sensitive line MO17 after drought treatment (SD). In this group, GO: 0008152 (metabolic process), GO: 0009987 (cellular process), GO: 0050896 (response to stimulus), GO: 0065007 (biological regulation), and GO: 0044699 (single-organism process) were the most significantly enriched GO terms in the BP category. In the MF category, GO: 0005488 (binding), GO: 0003824 (catalytic activity), and GO: 0005215 (transporter activity) were most significantly enriched (Table S1, Sheet 2).

Group C represents the 52 expressed genes that were shared by the tolerant and sensitive lines after drought treatment. In this group, GO: 0008152 (metabolic process), GO: 0009987 (cellular process), GO: 0044699 (single organism process), and GO: 0065007 (biological regulation) were the most significantly enriched GO terms in the BP category, whilst GO: 0005488 (binding) and GO: 0003824 (catalytic activity) were prominent in the MF category (Table S1, Sheet 3).

### 2.4. Differential Gene Expression Analysis 

The software package Cuffdiff [35] was used to explore differentially expressed genes (DEGs) between different treatments. Generally, at a standard fold change of less or equal to four (≤4) and false discovery rate (FDR < 0.001), both up- and down-regulated genes are expressed when plants are exposed to drought, in either treatment (before or after drought) or line type (tolerant or sensitive) [36]. Herein (at log2 FC, <0.001 FDR), before drought treatment, we identified a total of 4331 (1964 up-regulated and 2367 down-regulated) genes to be differentially expressed between the tolerant and sensitive lines (SC_TC in Table 3; Table S2).

**Figure 2:**
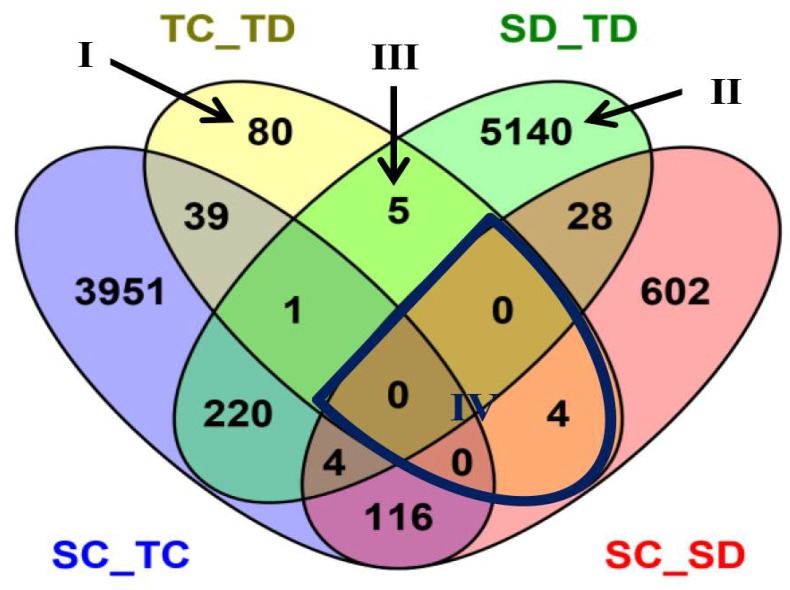
Venn diagram showing the profile of differentially expressed genes (DEGs). The differentiations were compared between inbred lines under each drought treatment, or between drought treatments in each inbred line. Drought treatments are labeled Control (C) and Drought (D). The tolerant line (YE8112) and the sensitive line (MO17) are labeled “T” and “S”, respectively. The four treatment-line biological samples are tolerant-control (TC), tolerant-drought (TD), sensitive-control (SC), and sensitive-drought (SD). Each compared combination is separated by an underscore (e.g., TC_TD). In the Venn diagram, the numbers of DEGs are shown across intersection areas among the compared combinations. In total we found 10,612 DEGs from all the areas. Four critical areas, labeled I, II, III, and IV, totally contain 5229 DEGs. Area I contains the tolerant treatment response DEGs, excluding others. Area II contains the line response under drought DEGs, excluding others. Area III contains both tolerance treatment response and line response under drought DEGs, excluding others. Area IV contains the treatment response DEGs within line.

Under water-deficit conditions, 5398 (2485 up-regulated and 2913 down-regulated) genes were observed in between the tolerant and sensitive lines (SD_TD in Table 3; Table S3). From these results, we further compared the differences (in DEGs) between the tolerant line YE8112 and sensitive line MO17. We identified 129 (49 up-regulated and 80 down-regulated) DEGs in the tolerant line (TC_TD) and 754 (329 up-regulated and 425 down-regulated) DEGs in the sensitive line (SC_SD), to be differentially expressed before and after drought treatment (Table 3; Tables S4 and S5). Between TC_TD and SD_TD, there were a total of five DEGs, which represent drought responsive DEGs specific to the tolerant line that were also differentially expressed between the tolerant and sensitive lines after drought treatment. In total, we found a total of 10,612 DEGs among the four comparison groups, which reflect the impact of lines or treatment (Figure 2).

Some of the combinations are more important than others in respect of drought tolerance. Area I represents 80 specific DEGs of TC_TD, that is, the specific drought responsive DEGs of the drought tolerant line YE8112. Of these DEGs, 31 were up-regulated and 49 were down-regulated (Table 4). Area II represents 5140 specific DEGs of SD_TD, that is, specific DEGs shared between the drought sensitive and drought tolerant lines after drought treatment. Of these, 2371 were up-regulated whilst 2769 were down-regulated (Table S6). Area III represents the five specifically shared DEGs between TC_TD and SD_TD, that is, drought responsive DEGs of the tolerant line that were also differentially expressed between the tolerant and sensitive lines after drought treatment. Of these, two were up-regulated and three were down-regulated in TC_TD comparison; whilst one was up-regulated and four were down-regulated in SD_TD comparison (Table 5). Area IV represents the four DEGs shared by TC_TD and SC_SD, that is, the common (overlapping) drought responsive DEGs within line. These DEGs showed differential expression in the two inbred lines after drought treatment, with two DEGs being up-regulated and the other two down-regulated in TC_TD. However, all the four DEGs were down-regulated in SC_SD (Table 6, Table S7). 

**Figure 3:**
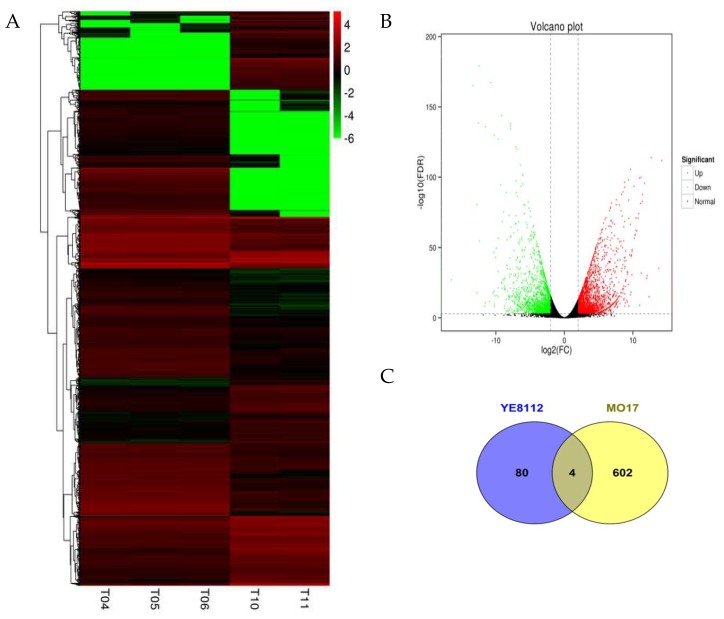
Clustering analysis of differentially expressed genes (DEGs) and number of DEGs in YE8112 and MO17. (**A**) Clustering analysis of DEGs in the inbred lines after drought stress treatment (SD_TD). The *x*-axis represents different samples. T04, T05, and T06 refer to the three replicates of tolerant line YE8112 drought stressed (TD); T10 and T11 refer to the two replicates of the sensitive MO17 drought stressed (SD); and the *y*-axis represents the differential genes expressed. The scale bar indicates up-regulated (red) and down-regulated (green) DEGs. The darker the color, the higher the expression, while the lighter the color, the lower the expression; G=genes of the same expression pattern are clustered together; (**B**) volcano plot showing the (log2 FC, −log10 FDR) expression of the DEGs in the SD_TD experimental comparison; (**C**) number of DEGs in drought stressed YE8112 and MO17 seedlings. The overlapping section of the Venn diagram shows the DEPs common to YE8112 and MO17 seedlings under drought stress conditions.

Clustering analysis of the DEGs of the SD_TD experimental comparison showed that, after drought stress exposure, DEGs were grouped into eight clusters, and more DEGs were down-regulated than up-regulated (Figure 3A). Additionally, analysis of the log_2_ fold changes of these DEGs showed that highest –log10 values were noted in the down-regulated DEGs than up-regulated DEGs (Figure 3B). Further, greater number of drought responsive DEGs were observed in the sensitive line MO17 than tolerant line YE8112 (Figure 3C).

### 2.5. DEGs Annotation and Functional Categorization

**Figure 4:**
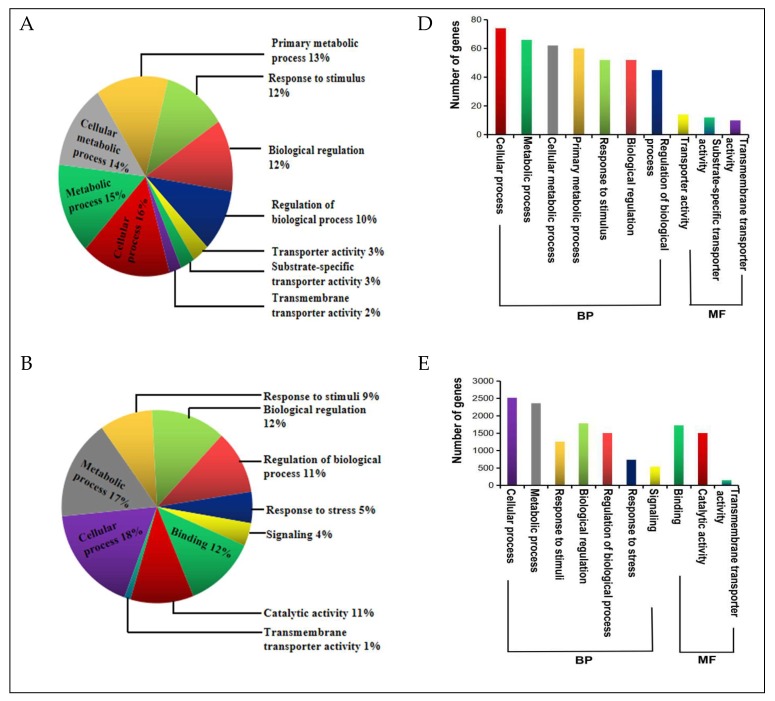

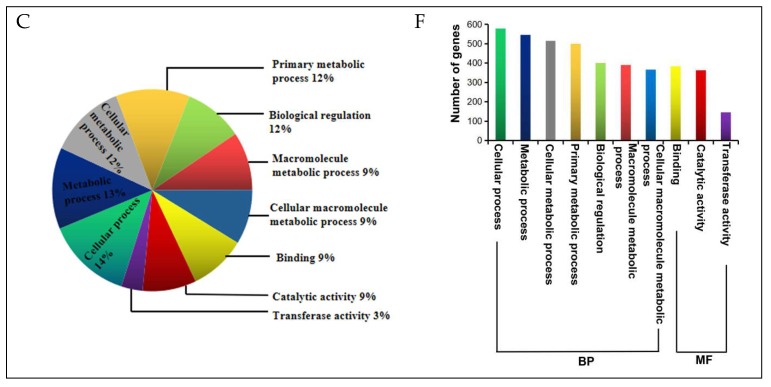
Gene ontology (GO) enrichment analysis of the DEGs (from the critical areas labelled I, II, and V in Figure 2) in specific level 2 GO terms. The GO analysis results shown here are for the top 10 GO terms from the biological processes (BP) and molecular functions (MF) categories combined, for (**A**) GO enrichment of DEGs corresponding to tolerant treatment response (TC_TD); (**B**) GO enrichment of DEGs corresponding to line response under drought (SD_TD); and (**C**) GO enrichment of DEGs corresponding to sensitive treatment response (SC_SD); (**D**–**F**) Number of DEGs enriched in each specific GO term in each experimental comparison (TC_TD, SD_TD, and SC_SD, respectively). Each area contained a background total of 80, 5140, and 602 DEGs, for TC_TD, SD_TD, and SC_SD, respectively.

All the DEGs got annotation and functional classification based on different databases (Table S8). To determine the broad biological functions of the DEGs from the four critical areas (labeled I–IV on Figure 2), we performed gene ontology (GO) functional annotation and categorization of those DEGs using the agriGO web-based program [37]. The GO analysis showed that a great number of DEGs were associated with biological processes (BP) and molecular functions (MF) under drought stress (Figure 4; Tables S9–S11).

For the Area I–IV specific DEGs, GO: 0009987 (cellular process); GO: 0008152 (metabolic process), GO: 0065007 (biological regulation), GO: 0006950 (response to stress), and GO: 0050896 (response to stimuli) were observed as the common and top most significantly enriched GO terms in the biological process (BP) category (Figure 4A–C). Within the molecular function (MF) group, GO: 005488 (binding); GO: 0005215/GO: 0005218 (transporter activity), and GO: 0003824 (catalytic activity) were most enriched among others (Figure 4A–C). These results suggest that the DEGs with these identified biological processes and molecular functions may be the key contributors to the drought response of the tolerant line YE8112 and the different drought responses between the two lines. For a comparative analysis, since the SC_SD specific DEGs were also enriched with the same common GO terms (Figure 4A–C), the significant difference in drought tolerance between the two inbred lines could be emanating from the difference in the number and specific types of genes enriched in each of those shared GO terms (Figure 4D–F), as well as the difference in the metabolic pathways in which those DEGs are involved. 

### 2.6. Differentially Expressed Genes Encoding Transcription Factors 

Transcription factors (TFs) are important regulators of drought stress [38]. Our analysis of the tolerant line YE8112 specific DEGs identified some transcription factor genes that were altered in response to drought stress. Two MYB-related genes, Zm00001d011297 (MYB-related TF35) and Zm00001d008808 (MYB-related TF24), were up-regulated in response to drought stress. Two WRKY genes, Zm00001d009698 (WRKY-TF20) and Zm00001d044162 (WRKY-TF64), were down-regulated. Meanwhile, two G2-like TF genes, Zm00001d013202 (G2-LIKE-TF8) and Zm00001d026542 (G2-LIKE-TF52), were down–regulated. On the other hand, one NAC gene, Zm00001d024268 (NAC-TF110), was up-regulated in response to drought stress (Table 4). Further, the TF gene Zm00001d051511 (PLATZ-TF7) was observed to be differentially expressed in the tolerant line YE8112 and in cultivar-specific comparison after drought stress (SD_TD); it was up-regulated in tolerant line YE8112, but down-regulated in SD_TD (Table 5). These TF genes could be the key contributors to drought stress tolerance in the tolerant maize inbred line. 

### 2.7. Metabolic Pathways Enrichment Analysis of the DEGs 

We further analyzed the functional involvement of the identified drought-responsive DEGs in various metabolic pathways by mapping them to the Kyoto Encyclopedia of Genes and Genomes (KEGG, available online: https://www.genome.jp/kegg/; accessed on 18 April 2018) database. Moreover, we performed significant KEGG pathway enrichment analysis of these DEGs by using the hypergeometric test [39]. By comparing the top twenty pathways that were most enriched in DEGs from different experimental comparisons, we discovered that nitrogen metabolism (five genes), carbon metabolism (4), biosynthesis of amino acids (3), and alanine and glucamate metabolism (3) were dominant in tolerant line YE8112 (TC_TD) (Figure S3A). 

In the SD_TD experimental comparison, plant hormone signal transduction (90 genes), phenylpropanoid biosynthesis (68), carbon metabolism (66), phenylalanine metabolism (57), and biosynthesis of amino acids (56) pathways had the greatest number of genes enriched (Figure S3B). However, the composition of the enriched KEGG pathways in the sensitive line MO17 (SC_SD) differed significantly, with ribosome (85 genes), carbon metabolism (73), plant hormone signal transduction (53), biosynthesis of amino acids (43), and photosynthesis (40) being the top most enriched pathways (Figure S3C). These results show that more DEGs were enriched in the pathways in MO17 than in pathways in YE8112, as we had noted previously [32]. 

**Figure 5:**
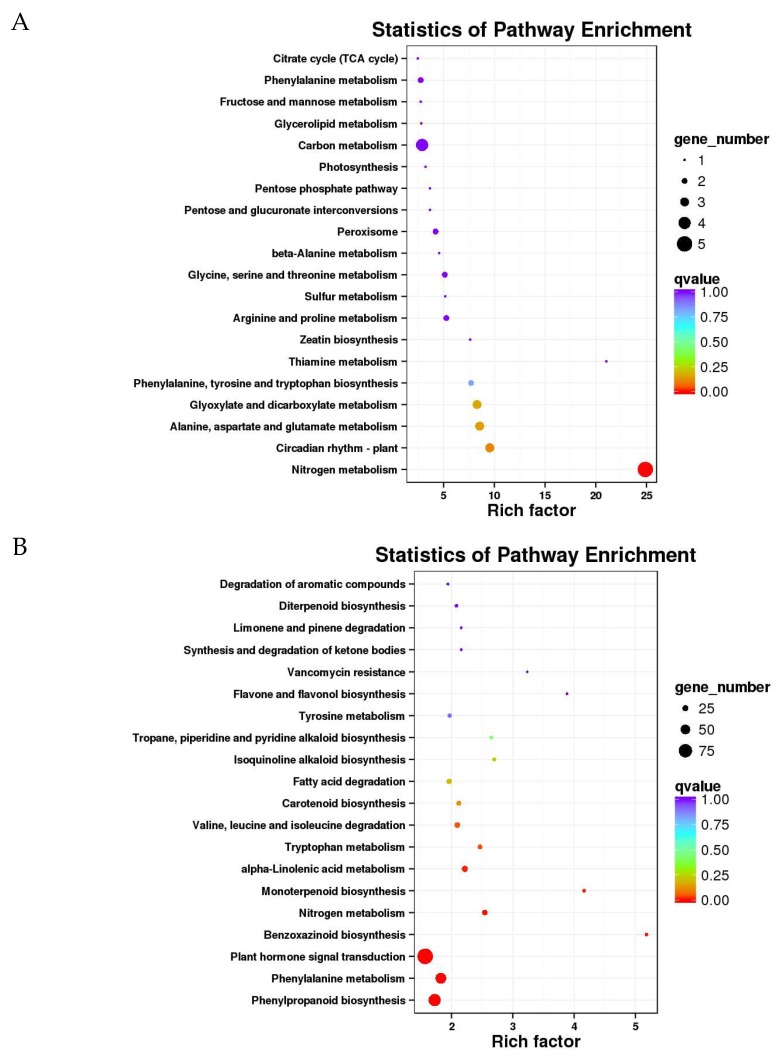

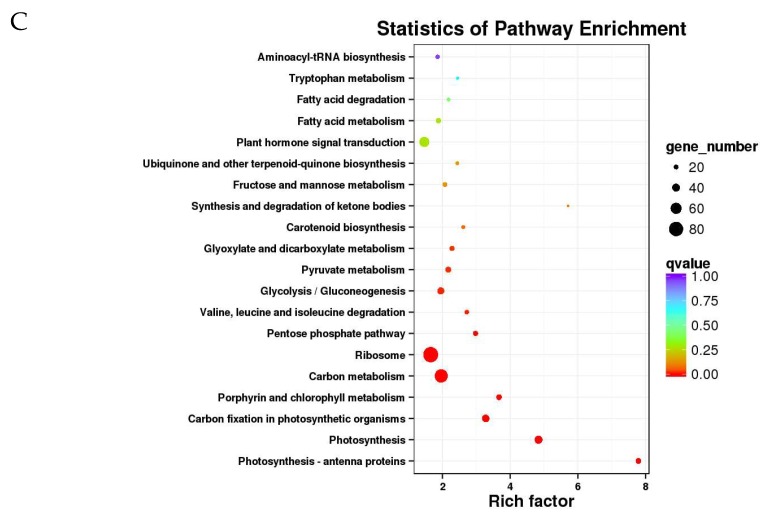
KEGG pathway enrichment analysis of the differentially expressed genes (DEGs). The sub-figures show the most significantly enriched pathways in (**A**) TC_TD; (**B**) SD_TD; and (**C**) SC_SD experimental comparisons, based on the hypergeometric test. The significance of the enrichment of the KEGG pathway is based on the Student’s *t*-test, *q* < 0.01. The color gradient represents the size of the *q* value; the color is from red to blue, and the nearer the red represents the smaller the *q* value, and the higher the significant level of enrichment of the corresponding KEGG pathway. The “rich factor” shows the ratio of the number of the DEGs to the total gene number in certain pathways.

Using a hypergeometric test, KEGG pathways that had a *q* value < 0.01 were considered to be significantly affected by drought stress. The results showed that the nitrogen metabolism pathway was the most highly enriched of the KEGG pathways in the tolerant line YE8112 (Figure 5A), whilst plant hormone signal transduction, phenylpropanoid biosynthesis, phenylalanine metabolism, and nitrogen metabolism pathways were highly enriched in the SD_TD comparison (Figure 5B). In contrast, ribosome, carbon metabolism, and photosynthesis related pathways were the most significantly enriched in the sensitive line MO17 (Figure 5C).

### 2.8. DEGs Related to “Response to Stress” and “Response to Stimuli” 

Our analysis of the DEGs that were enriched in each level 2 GO term showed that, among the tolerant line YE8112 specific DEGs (listed in Table 4), 28 (Table S12) were significantly enriched in GO term “response to stress” (GO:0006950). Further analysis of these DEGs also showed that they were enriched in GO term “response to stimuli” (GO: 0050896), and they were also found in the SD_TD experimental comparison (Table S10). Among these genes were *Zm00001D023425* (*CRINKLY4*), *Zm00001D019983* (BRI1-like receptor kinase 1), *Zm00001D034345* (ferredoxin NADP reductase 1), *Zm00001D018738* (pathogenesis related protein 4), *Zm00001D011473* (potassium channel 5), *Zm00001D024546* (late hypocotyl elongation protein), *Zm00001D044162* (*WRKY 64*), *Zm00001D028164* (Sulfate transporter 2.2), *Zm00001D044642* (calcineurin B-like-interacting kinase), and *Zm00001D033047* (SPX domain-containing protein 3) (Table S10). These genes are involved in stress signal perception and transduction, nutrient and water uptake, and cell elongation under stress conditions, and; thus, are considered vital in drought stress tolerance in maize seedlings. 

### 2.9. Validation of DEGs by Quantitative Real-Time PCR (qRT-PCR) 

**Figure 6:**
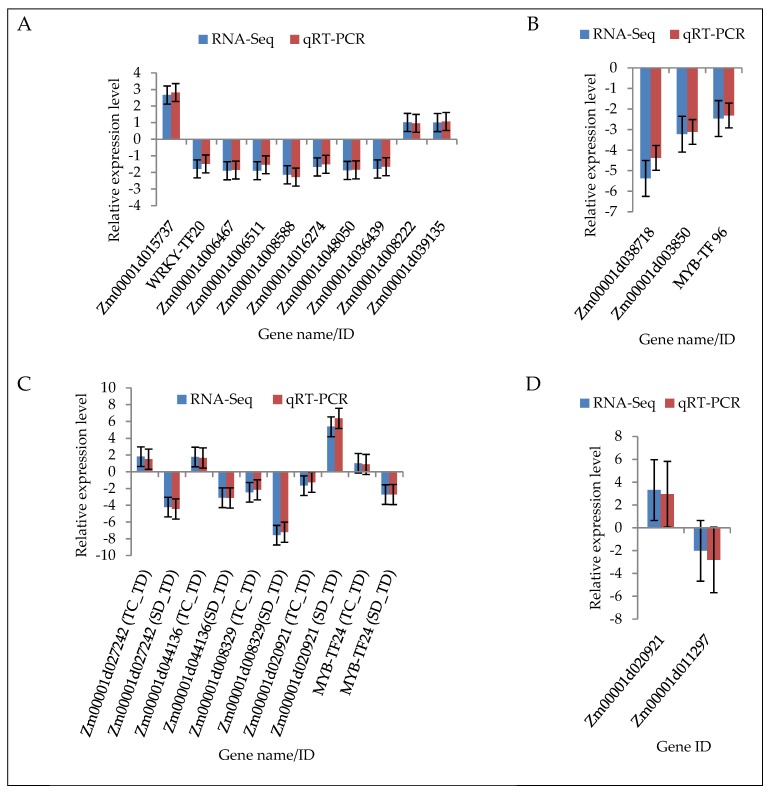
qRT-PCR validation of the RNA-seq data of the 20 randomly selected maize seedling leaf differentially expressed genes (DEGs). (**A**) TC_TD specific DEGs; (**B**) SD_TD specific DEGs; (**C**) DEGs shared between TC_TD and SD_TD; and (**D**) DEGs specific to SC_SD. The *y*-axis represents the gene relative expression levels (fold changes) in the real-time PCR analysis and fold changes in the RNA-seq data. All the genes with negative values of expression level means that they were down-regulated in response to drought stress. Maize gene *GAPDH* (accession no. X07156) was used as the internal reference. Error bars represent the SE (*n* = 3).

To confirm the accuracy of the RNA sequencing (RNA-seq) results, a sample of 20 DEGs were randomly chosen for quantitative real-time PCR (qRT-PCR) analysis. The gene specific primers (Table S13) used for qRT-PCR analysis were designed using Primer Premier 5.0 software (Premier Biosoft International, Palo Alto, CA, USA). The results of the qRT-PCR analysis validated our findings based on RNA-seq data. Precisely, for all the 20 DEGS, the PCR expressions were consistent with the RNA-seq data, that is, the patterns of the RNA-seq expression on all the sampled genes were replicated by the qRT-PCR approach (Figure 6; Table S14). A correlation coefficient (*R*^2^) (of the fold changes between qRT-PCR and RNA-seq) of 98.86% was obtained (Figure S4), endorsing that our RNA-seq data was reliable. 

## 3. Discussion

In order to respond to drought stress, plants have evolved complex adaptive mechanisms at the physiological, biochemical, and molecular levels [13,40]. However, the molecular mechanisms underpinning this phenomenon have remained elusive despite recent advances in molecular biology approaches [41]. Therefore, in the current report, we have employed an RNA-seq-based approach to perform a comprehensive comparative transcriptome analysis of two contrasting maize (drought-tolerant YE8112 and drought-sensitive MO17) inbred lines to identify key regulatory genes and metabolic pathways involved in maize drought stress tolerance. In addition, we have conducted some physiological analyses to support the RNA-seq data. Further, functional validation by qRT-PCR analysis corroborated the differential expression of these identified genes. Our findings provide further insights into the elucidation of drought stress tolerance in maize, as well as providing a basis for further downstream analyses of the identified individual specific genes.

### 3.1. Clear Divergence Exist Between Inbred Lines YE8112 and MO17 in Their Drought Stress Responses

Tolerant line YE8112 seedlings exhibited higher leaf relative water content than sensitive line MO17 seedlings under drought stress conditions (Table 1). Additionally, analysis of the proline and POD contents of the two genotypes revealed that YE8112 seedlings always accumulated greater amounts of the protective osmolyte and antioxidant enzyme than MO17 seedlings under water limited conditions (Table 1); this was in agreement with the previous report [42]. Furthermore, tolerant line YE8112 maintained lower MDA content than sensitive line MO17, both under control and water-deficit conditions (Table 1). 

It is well known that drought stress physiologically represses plant growth by having less biomass accumulation per unit area and lower photosynthesis rates [43]. At the cellular level, when plants suffer from drought stress, the decreased cellular volume causes crowding of cytoplasmic components. The chances for molecular interactions would increase, effected by the increasingly viscous cell contents, consequently resulting in protein denaturation and membrane fusion [44]. As a response, and in order to avoid these deleterious interactions, plants maintain the membrane stability and stabilize protein structures by employing several measures, including decreasing photosynthesis rate through leaf rolling, stomata closure, cell turgor maintenance, and osmotic adjustment [45,46].

Here, our results showed that, in tolerant line YE8112, proline content and POD activity increased under water stress condition. Proline reduces osmotic potential; then, the plants could maintain cell turgor pressure and growth by changing the turgor-raising potential of the existing cells [47]. Peroxidases act as the first line of cell defense by scavenging and detoxifying ROS-generated H_2_O_2_ [48], hence better cushion of cells possessing enhanced POD activity. Moreover, the tolerant line YE8112 cells’ enhanced ROS quenching competency resulted in greater cell membrane integrity and, consequently, better drought stress endurance than sensitive line MO17 [3,32,42]. 

Further, the results from RNA-seq analysis showed that the two genotypes’ responses to drought stress were quite different. Cultivar-specific pairwise analysis showed that, after drought treatment (at log2 FC, <0.001 FDR), drought-tolerant YE8112 had relatively lower DEGs than drought-sensitive MO17 (Table 3). Under water limited conditions and compared to sensitive line MO17, tolerant line YE8112 sustained greater leaf relative water and proline contents (Table 1), consequently resulting in relatively lower stress at the cellular level. This explains why tolerant line YE8112 exhibited a limited transcriptome response. Similarly, previous studies [3,9] have observed higher number of DEGs in sensitive than tolerant maize inbred lines after drought and freezing treatments. Taken collectively, our results have shown that divergent responses to drought stress exist between YE8112 and MO17 inbred lines, and that there was coherence between the physiological characterization and transcriptome profiling data of the two lines.

### 3.2. Stress Signal Transduction and Protein Kinases under Drought Stress Conditions

Stress perception is the first step to ensure plant survival to abiotic stress exposure [49]. The stress is first perceived by the receptors present on cell membranes. The signals are then transduced downstream and this results in the generation of secondary messengers including K^+^, Ca^2+^, sugars, ROS, cyclic nucleotides, and inositol phosphates [4]. The secondary messengers further modulate the intracellular calcium level. This perturbation in the cytosolic Ca^2+^ level is sensed by calcium binding proteins known as Ca^2+^ sensors [50]. These secondary messengers will eventually trigger the corresponding signaling pathways to transduce the signals [51]. Central to the signal transduction machinery are protein kinases and phosphates that mediate protein phosphorylation and dephosphorylation, respectively [14]. The calcium-dependent protein kinases (CDPKs) and mitogen activated protein kinases (MAPKs) pathways are vitally involved in plant abiotic stress responses [52,53]. At the end of the signal transduction cascade, transcription factors (TFs) are modulated by the protein kinases or phosphates, consequently effecting corresponding responses to the downstream drought responsive genes [38].

In the current study, a set of genes implicated in signaling and cell communication, such as K^+^ channel (*Zm00001d011473*) and Cl^–^ channel (*Zm00001d015700*) were up-regulated by 2.59 times and down-regulated by 1.08 times, respectively (Table 4). Several protein kinases were also identified to be differentially expressed in the tolerant line YE8112, including *CALCINEURIN_B-LIKE10* (CBL, *Zm00001d044285*) and protein kinase superfamily protein (*Zm00001d017374*) that were up-regulated in response to drought stress. Calcineurin B-like protein interacting kinases (CIPKs) are a group of Ser/Thr protein kinases that mediate calcium signals [54]. In rice (*Oryza sativa* L.), CIPK gene *OsCIPK31* has been involved in germination and seedling growth under abiotic stress conditions [54]. Further, ZmCIPK8, a CBL-interacting protein kinase, has been crucially involved in regulating maize drought stress response [55]. From this discussion, we can infer that these protein kinases play pivotal roles in the drought stress response mechanism. However, the down-regulation of some protein kinase related genes (*Zm00001d021135* and *Zm00001d044642*) herein may imply the complexity of the stress signaling network, as various elements interact to effect drought stress responses.

### 3.3. Transcription Factor (TF) Related Genes Are a Critical Component of Drought Response Machinery 

Drought stress response is controlled by a complex gene regulatory system [56]. Transcription factors (TFs) have been designated as master regulators of abiotic stresses, including drought, participating as key controllers of multiple downstream stress-responsive genes [38]. Most of these reported TFs belong to MYB, NAC, WRKY, zinc finger, AP2-EREBP, and bZIP families, and their analysis has played a very important part in stress response research [13,14,22]. Herein, several TF related DEGs were identified in the tolerant line YE8112. There were a set of transcription factors, including WRKY (2), MYB-related (2), GARP-G2-like (2), and NAC (1) (Table 4). Among these, two *WRKY* genes were down-regulated, two *MYB*-related genes were up-regulated, and two *GARP-G2*-like genes were down-regulated, whilst one *NAC* gene was up-regulated in response to drought stress (Table 4). Furthermore, numerous transcriptional factors were up-regulated in SD_TD comparison, especially the AP2-EREBP, WRKY, bZIP, MYB, bHLH, NAC, and G2-like TFs (Table S6). 

Previously, in a transcriptome study to identify 20% PEG 6000—induced osmotic responsive genes in maize seedlings, Shan et al. [13] observed that, among the up-regulated genes, the *NAC*, *MYB,* and *DREB* transcriptional factor genes were prominent. Shi et al. [57] identified twelve transcription factor related DEGs, among them five up-regulated NACs, three up-regulated MYBs, and two down-regulated MYBs were expressed in foxtail millet (*Setaria italic* L.) drought stress response. Interestingly, among the four overlapping DEGs in the current study, gene *Zm00001d014863* (*MYB-related-TF96*) was up-regulated in tolerant line YE8112, but down-regulated in sensitive line MO17 (Table 6). A previous study by Bianchi et al. [58] revealed that amongst the TFs genes, the WRKY genes were redundant, largely exhibiting down-regulation under drought stress. However, the NAC factor was up-regulated and played diverse roles in drought stress response. Additionally, Zhang et al. [59] observed several genes with expression tightly coupled to plant water potential, including eight NACs, eight MYBs, six AP2/EREBPs, six bZIPs, five HDs, four bHLHs, and other TFs, suggesting their involvement in *Medicago truncatula* drought adaptation responses. Furthermore, in a study by Yan et al. [60], WRKY TFs were identified as the key drought response elements. More recently, in a study in foxtail millet (*Setaria italic* L.) by Shi et al. [57], several transcriptional factors were identified to be differentially expressed in tolerant line M79 in response to drought stress, including 20 NAC, 14 WRKY, and five DREB, among others. Taken together, we can conclude that the differential expression of the *WRKY*, *MYB-related*, *GARP-G2*-like, and *NAC* TF genes crucially contribute to the drought stress tolerance of maize inbred line YE8112, through these TF genes interacting with each other in complex networks.

### 3.4. Enhanced Cellular Redox Homeostasis Is Essential for Plants to Tolerate Drought Stress 

When plants are subjected to drought stress, there is a rapid and transient production of ROS, which can damage cellular components and structures [43]. In response, the drought stressed plants achieve the re-establishment of the cellular redox balance and homeostasis by altering their metabolism through various means [19]. These include the production of osmoprotectants (such as proline) that reorganize proteins and cellular components, and maintain cell turgor by osmotic adjustment and modifying the antioxidant system [50]. Additionally, the drought responsive genes, such as the late embryogenesis abundant (LEA) proteins, dehydrins, heat shock proteins (HSPs), and other molecular chaperons are modulated in response to stress [14,16]. In the current study, our physiological characterization of the inbred lines has confirmed higher accumulation of proline and peroxidase (POD) in the tolerant line YE8112 than sensitive line MO17 under drought stress (Table 1). Further, our transcriptome results have observed several enzymes, proteins, or genes implicated in redox homeostasis to be up-regulated in the SD_TD experimental comparison group (labeled Area II on Figure 2). These include thioredoxin reductase (*Zm00001d053118*, *Zm00001d052034*), peroxidase (*Zm00001d052336*), betaine aldehyde dehydrogenase (*Zm00001d050339*), FAD/NAD (P) binding oxidoreductase family protein (*Zm00001d052784*), and dehydrins (DHNs) (*Zm00001d051420*, *Zm00001d051263*), among others. Moreover, drought responsive genes were up-regulated, including LEA proteins (*Zm00001d052774* and *Zm00001d050863*), HSPs (*Zm00001d051607* and *Zm00001d052809*), heat stress transcriptional factor B-2b (*Zm00001d052738*), and chaperons and chaperonins (*Zm00001d051342 Zm00001d052001*, *Zm00001d053667*, *Zm00001d053279* and *Zm00001d052101*), among others (Table S6).

The chloroplast thioredoxin (THX) systems constitute a critical component of the redox network, with thioredoxin reductase (TRs) functioning in restoring redox homeostasis by reducing the oxidization. These versatile plant chloroplast TRs systems comprise two reductases, dependent on ferredoxin and NADPH, respectively [61]. The proline osmolyte and POD antioxidant enzyme detoxify the damaging ROS, hence protect the cells from the oxidative damage; their roles have been extensively discussed in previous reports [62,63]. The LEA proteins are known to accumulate during late embryogenesis and are induced in vegetative tissues by various abiotic factors (including drought stress) that cause dehydration. They are activated to prevent intracellular water loss [16]. Further, the DHNs, a distinct group of LEAs, are ubiquitous and responsive to ABA, that is, their expression is increased by the phytohormone abscisic acid [64], and they have been observed to play a critical role in plant stress responses [16]. Drought stress induced constitutive expression of HSPs and chaperons helps in cushioning intracellular proteins against denaturation, thereby preserving their functional confirmation [19]. Taken collectively, this discussion can reveal that enhanced cellular redox homeostasis is essential for plants to tolerate drought stress.

### 3.5. Carbohydrate Metabolism and Cell Growth Promotion Are Vital for Seedlings Survival under Drought 

Carbohydrate (CHO) metabolism is at the center of bio-molecular metabolism; substrates participating in CHO breakdown offer the important saccharides and energy that the cell requires [3,20]. Additionally, the essential expression profile changes effected to the CHO metabolism related genes may induce positive feedback and corresponding adjustment in plants adapting to drought stress [3]. In the current study, our GO term enrichment analysis (under the biological process (BP) category) revealed that GO:0008152 (metabolic process), GO:0009987 (cellular process), and GO:0065007 (biological regulation) were the top most enriched terms in the tolerant line YE8112 (TC_TD) specific DEGs (Table S9). Further analysis of these DEGs showed that 37 genes were overlapping in these three top GO terms. We then considered these genes central to better drought stress tolerance of inbred line YE8112. Among these 37 DEGs, some genes involved in starch synthesis, such as granule-bound starch synthase (*Zm00001d027242*) and beta-amylase (*Zm00001d029154*) were up-regulated in the tolerant line YE8112 after drought stress treatment (Table 4); this contributed to enhanced CHO reserves. 

Cell-wall metabolism related genes were also up-regulated in the tolerant line YE8112 in response to drought stress exposure. Among these were *BETA EXPANSIN 7* (*Zm00001d029906*), *TRICHOME BIREFRINGENCE-LIKE 20* (TBL20, *Zm00001d017918*), and putative O-Glycosyl hydrolase (*Zm00001d038049*). Beta-expansins, together with alpha-expansins, are cell wall proteins that have become widely acknowledged as key regulators of cell wall modifications, particularly during tissue elongation [65,66]. TBL20 gene participates in secondary wall cellulose synthesis and deposition, probably by influencing the esterification form of pectic polymers [67]. In *Oryza sativa* L., the *O*-Glycosyl hydrolases have been implicated in plant adaptation processes, including response to biotic and abiotic stresses, phytohormones activation, cell wall remodeling, and lignification [68]. Taken collectively, the up-regulation of cell-wall-related genes could indicate that cell-wall adjustment may be a protective strategy of tolerant line YE8112 against moisture deficit, and; thus, an indispensable adaptive response to drought stress in maize. This observation resonated well with previous findings [3,69]. 

Furthermore, proteins involved in cell growth promotion were up-regulated in response to drought stress, including late hypocotyl elongation protein ortholog 1 (*LHY,* *Zm00001d024546*) and protein light-dependent short hypocotyls 5 (*LSH5*, *Zm00001d046998*) (Table 4). In plants, the *LHY* gene is involved in circadian rhythms coordination, a critical process modulating leaf movements, stomata opening, hypocotyl elongation, flower initiation, and gene expression [70]. The *LSH5* gene has been designated a probable transcription regulator that coordinate plant development in *Arabidopsis thaliana*, through promoting cell growth in response to light (www.uniprot.org/uniprot/, accessed on 5 November 2018). The up-regulation of these genes may suggest that the tolerant maize line seedlings battle drought stress by prioritizing cell growth in the hypocotyl so that they get well-established in the shortest possible time. Overall, enhanced carbohydrate metabolism could have resulted in increased energy production and cell elongation, which assisted drought stressed plants to maintain normal growth under adverse conditions, whereas cell wall remodeling could have helped the cells to conserve water, thereby helping YE8112 seedlings to better adapt to water deficit conditions [69]. 

### 3.6. Protein Ubiquitination Plays a Significant Role in Drought Stress Response Regulation

Protein ubiquitination has been widely acknowledged as a central regulator of stress responsive transcription factors and other regulatory proteins, effectually contributing to abiotic stress adaptation [69,71,72]. Protein ubiquitination, via the ubiquitin-proteasome system (UPS), acts as a signal for selective protein degradation [73]. The UPS functions in the efficient perception of abiotic stresses and modulation of the downstream responses. It achieves this through suppression of certain stress signaling pathways during normal non-stress conditions, blocking negative stress signal response regulators, or attenuating the signal pathway once the stress has terminated [74]. In the current study, among the tolerant line YE8112 specific DEGs, several genes encoding proteins involved in ubiquitination were up-regulated, including Probable BOI-related E3 ubiquitin-protein ligase 2 (*Zm00001d003850*), RING-H2 finger protein ATL3F (*Zm00001d040639*) and cysteine protease 1 (*Zm00001d013261*) among others (Table 4). Moreover, among the SD_TD specific DEGs, there were numerous (above 90) ubiquitin-related and several RING zinc finger domain super family protein related genes that were up-regulated in response to drought stress (Table S6). The E3 ubiquitin ligases have been identified as key elements in nuclear protein homeostasis during plant stress responses. Particularly, they participate in transcription-dependent resistance to high temperature and drought stress [75]. The RING-H2 finger protein and cysteine proteases play a critical housekeeping function by removing damaged, abnormal, or misfolded proteins, as well as controlling the accumulation of certain regulatory proteins during abiotic stress [76]. From this discussion, we can herein infer that the protein turnover mechanism effected by these up-regulated protein ubiquitination-related genes is vital for coordinating the cellular crosstalk between stress and hormone signaling, effectively contributing to drought stress adaptation.

### 3.7. Overlapping Drought Responsive DEGs between Inbred Lines under Drought Stress 

Our key observation that the two overlapping genes, viz., *MYB-related TF96* and *pyrophosphate fructose-6-phosphate 1-phosphotransferase subunit alpha 1* (*PFP ALPHA 1*) were up-regulated in tolerant line YE8112, but down-regulated in sensitive line MO17 (Table 6), offered us some clue as to some of the key differences in drought tolerance between the two lines. MYB transcription factor (TF) constitutes one of the largest TF families that coordinate plant defense responses to various stresses, phytohormone signaling, and various metabolic processes [77]. Specifically, MYB96 participates in cuticular wax biosynthesis under drought stress, consequently leading to enhanced plant tolerance to drought by reducing stomatal opening [78]. Further, in *Arabidopsis thaliana*, MYB96 has been identified as a molecular link that mediates ABA-auxin cross talk regulating lateral root growth under drought stress conditions, thereby offering an adaptive strategy under such conditions [79]. Our results suggest that, in response to drought stress, the tolerant line YE8112 activated the MYB TF gene to modulate ABA-signaling, the defense response, and cell-wall modification. The invigorated ABA signaling cascade enhanced the seedlings’ stress signal perception and transduction, as well as lateral root growth for better scavenging of water and nutrients. Enhanced cell-wall modification, through differentiation and stomatal regulation, offered the YE8112 seedlings better adaptation to drought stress as compared to the sensitive line MO17. 

*PFP ALPHA 1* is the regulatory subunit of pyrophosphate fructose-6-phosphate 1-phosphotransferase (PFP), and is involved in the glycolysis pathway. PFP is stimulated by Fru-2,6-P_2_ allosterically [80]. Fru-2,6-P_2_ is a signal metabolite, and in *Nicotiana tabacum* L. leaf tissues, it critically regulates photosynthetic carbon metabolism; contributing to the coordination of sucrose synthesis, as well as to the control of photosynthate partitioning between sucrose and starch [81]. This may suggest that PFP and Fru-2,6-P_2_ functions overlap in plant stress responses [82]. In *Daucus carota* L. plants, PFP has been implicated in mobilization of energy reservoirs upon cold and drought stresses, by promoting the re-synthesis of transportable sucrose via gluconeogenesis from accumulated starch in taproots [81]. Here, we submit that the up-regulation of *PFP ALPHA 1* in drought stressed YE8112 seedlings may have enhanced better stress signal perception, carbon metabolism, and efficient CHO partitioning, as well as energy mobilization to different cell and plant parts. 

### 3.8. Metabolic Pathways Significantly Enriched under Drought Stress Conditions 

Analysis of metabolic pathways enrichment contributes to the systems-biology approach of understanding stress biology of plants [83]. Here, our KEGG pathway enrichment analysis revealed that the nitrogen metabolism pathway was the most significantly (*p* < 0.01) enriched in the tolerant line YE8112 under drought stress conditions (Figure 5A). Interestingly, the same pathway was also significantly enriched in the SD_TD specific DEGs, suggesting that it plays a key role in drought stress response. Nitrogen metabolism pathway is the most basic and important physiological metabolic process during plants’ growth period; it directly influences the formation of cellular components and regulation of cellular activities, as well as the transformation of photosynthetic products, mineral nutrient absorption, and the process of protein synthesis [43,84]. Moreover, inorganic-to-organic nitrogen conversion process is a vital protective mechanism employed by plants against ammonia toxicity [84]. A transcriptome study on *Pugiomium cornutum* L.—a xerophytic plant species adapted to sandy and desert conditions—revealed the nitrogen metabolism pathway is significantly correlated to drought response [36]. Further, the nitrogen metabolism pathway has been revealed as a key response pathway in salt stress [84], suggesting its importance in abiotic stress responses. 

Plant hormone signal transduction, phenylpropanoid biosynthesis, and phenylalanine metabolism pathways were the top most significantly enriched in the SD_TD specific DEGs (Figure 5B). Plant hormone signaling pathways participate in abiotic stress adaptation through various manners, including ubiquitin-mediated proteolysis [72] or ABA-mediated response [13]. Phenylalanine ammonia lyase (PAL) takes part in the initial stage of phenylpropanoid metabolism, which is the first step of the secondary metabolites (flavonoids, phenylpropanoids, and lignin) biosynthesis [85]. The phenylpropanoid metabolism pathway serves to provide these secondary metabolites, which then contribute to stress tolerance [72]. A coordinated reaction of the genes and pathways involved in secondary metabolite biosynthesis is; thus, considered vital to improved drought stress tolerance in plants.

Comparatively, in the sensitive line MO17, the ribosome pathway was the most significantly enriched in response to drought stress (Figure 5C). Ribosomes are the site for protein synthesis, one of the fundamental biological processes that is influenced by abiotic stress [72], and hence the pathway was enriched. However, the drought susceptibility of the sensitive line MO17 may be emanating from its nitrogen metabolism incompetency, probably due to inefficient nitrate reduction or poor photosynthetic products transformation and mobilization. Additionally, MO17 seedlings’ lavish production of redundant proteins (as evidenced by ribosome pathway getting enriched) may have weakened plants’ thriving ability under drought stressed conditions. Contrastingly, it may be probable that the tolerant line YE8112 may have decreased the production of redundant proteins as an adaptive response to conserve energy and enable the plants to overcome the effects of the imposed stress [86,87]. We refer you to Figure S5 for a full pictorial view of the top most significantly enriched pathways in the two inbred lines. 

### 3.9. Proposed Molecular Model of Maize Seedling Drought Stress Tolerance

**Figure 7:**
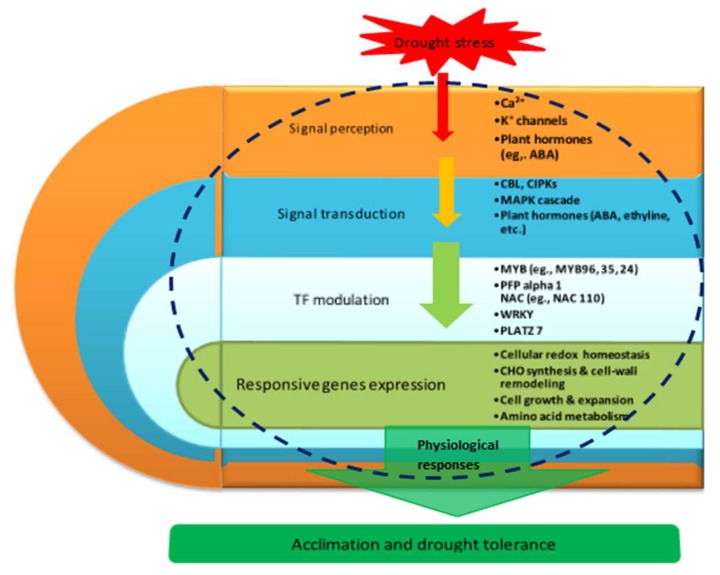
Schematic molecular model of maize seedling drought stress tolerance. This model was developed based on our key putative components of drought response identified in this study, supported by previously described schemes of plant abiotic stress response pathways/networks [9,38,72]. The blue dotted enclosure signifies the molecular interactions occurring in the cellular environment. The down-ward pointing arrows show the connections between the components of the drought response network, from stress signal perception through signal transduction to transcriptional regulation of gene expression, until physiological and acclimation mechanisms are instituted in whole plants to effect drought tolerance. Key to abbreviations: Ca^2+^, calcium signals receptors; K^+^, potassium channels signal receptors; ABA, abscisic acid; CBL, calcineurin B-like; CIPKs, CBL-interacting protein kinases; MAPK, mitogen-activated protein kinases; TF, transcription factor; MYB, myeloblastosis oncogene; *PFP alpha 1*, pyrophosphate fructose-6-phosphate 1-phosphotransferase subunit alpha 1; NAC, (**NAM**, **ATAF**_1_/_2_, and **CUC**_2_) domain proteins; WRKY, family denoted by protein domain composed of a conserved WRKYGQK motif and a zinc-finger domain; PLATZ, plant AT-rich sequence and zinc binding protein 1; CHO, carbohydrates.

Based on our main findings of the key drought responsive DEGs and their associated pathways/networks, and the relevant published citations contained in this study, we have developed a molecular model for drought stress tolerance in maize seedlings as shown in Figure 7.

## 4. Materials and Methods 

### 4.1. Plant Materials and Drought Stress Treatment 

The two maize inbred lines contrasting in drought stress sensitivity (tolerant YE8112 and sensitive MO17) used in this experiment were provided by our lab (North China Key Laboratory for Crop Germplasm Resources of Education Ministry, Hebei Agricultural University, Baoding, China). Detailed information on the selection of these inbred lines, seedlings establishment, and drought stress treatment is contained in our most recent study [32]. Flag leaves from the control and drought stress treated plants were collected seven days post drought treatment exposure for transcriptomic analysis. The collected samples were immediately liquid-nitrogen frozen prior to storage under an extreme low temperature (−80 °C) conditions awaiting subsequent analyses; each treatment had three replications.

### 4.2. Physiological and Phenotypic Characterizations

As already presented in our most recent paper [32], we analyzed some physiological and phenotypic responses of both inbred lines to drought treatment (control and water-deficit) conditions. Seedling leaf relative water content (RWC) was estimated as per the protocol of Galmés et al. [88]. Additionally, leaf peroxidase (POD) activity was estimated by Han’s guaiacol method [89]. The thiobarbituric acid (TBA) method [90] was used to determine the level of lipid peroxidation (MDA content) in the leaf samples, whereas proline content was measured using nin-hydrin as per the protocol of Bates et al. [91]. Phenotypic responses were analyzed by visual observation.

### 4.3. Total RNA Extraction, cDNA Library Construction, and Transcriptome Sequencing

Maize inbred lines YE8112 (drought-tolerant) and MO17 (drought-sensitive) were grown according to the method described [32]. Total RNA of the leaf samples (control and drought-exposed second-upper-most-leaf samples), which had been stored at −80 °C, was isolated using Trizol reagent (Invitrogen, Carlsbad, CA, USA) following the manufacturer’s protocols. To remove genomic DNA, the RNA was purified and concentrated using a RNeasy column (QIAGEN, Pudong, Shanghai, China). RNA degradation and contamination (integrity) was monitored on 1% agarose gels. RNA purity and concentration were checked using the NanoDrop 1000 spectrophotometer (NanoDrop Technologies Inc., Wilmington, DE, USA). The cDNA library construction and sequencing (on an Illumina Hiseq Xten platform, San Diego, CA, USA) were conducted by Novogene Bioinformatics Technology Co., Ltd. (Beijing, China). 

### 4.4. Sequencing Reads Processing, Mapping, and Gene Expression Quantification

Initial processing of raw data/reads (FASTQ format) was achieved through in-house PERL scripts. Basically, this stage involves trimming raw data by removing some reads containing adaptor sequences, ploy-*N*-containing reads, and low-quality sequences in order to obtain clean data (clean reads). Simultaneously, quality (Phred quality) scores for assessing sequencing accuracy were calculated, including Q20 (99% base call accuracy), Q30 (99.9% base call accuracy), as well as the GC content and sequence duplication level of the clean data. Consequently, high quality clean data was used in all the subsequent analyses. Qualified clean reads were then mapped to the maize reference genome sequence (B73 RefGen_v3) using a spliced aligner Tophat 2.0.12 software [92]. Only reads with a perfect match or one mismatch were further analyzed and annotated based on the reference genome. In addition, HTSeq v 0.6.1 software [93] was used to count the read numbers that were mapped to each gene. Quantification of gene expression levels were estimated by reads per kilobase of transcript per million mapped reads (RPKM = otal exon reads/mapped reads in million × exon length in kb) for each gene and log2 transformed [33]. After the aligned reads had been analyzed using Cufflinks v2.2.1 [34], the assembled transcripts were further filtered using the FPKM value > 1.

### 4.5. Functional Annotation of Gene Transcripts

For functional annotation, the assembled gene transcripts that might search against the following public databases: non-redundant protein sequence database (Nr) (https://www.ncbi.nlm.nih.gov/); Swiss-Port (a manually annotated and reviewed protein sequence database) (https://web.expasy.org/docs/swiss-prot); Clusters of Orthologous Groups (COG) (https://www.ncbi.nlm.nih.gov/COG/); and the Kyoto Encyclopedia of Genes and Genomes (KEGG) (http://www.genome.jp/kegg) were scavenged using the BLAST (basic local alignment search) search program [94]. Gene ontology (GO) terms were assigned to each gene transcript using the agriGO (http://systemsbiology.cau.edu.cn/agriGOv2/#) web-based program [37]. The threshold for significantly enriched GO terms was set at *p*-value < 0.05, FDR < 0.001.

### 4.6. Differentially Expressed Genes (DEGs) Library Construction and Differential Analysis

The differentially expressed genes (DEGs) sequencing libraries were constructed as per the expected standards of the transcriptome sequencing libraries. Anders’ [95] DESeq R software package (1.10.1) was employed to execute gene differential expression analysis of two experimental groups/conditions, based on the negative binomial distribution model [96]. The method uses student *t*-test to calculate the *p*-values, and the threshold level was set at *p*-value ≤ 0.05. The resulting *p*-values were adjusted using the Benjamini and Hochberg’s [97] approach for controlling the false discovery rate. Finally, DEGs with an FDR < 0.001 |log2 FC| were assigned as differentially expressed. 

### 4.7. Gene Ontology (GO) Enrichment and KEGG Pathway Enrichment Analyses

To study the biological significance of the DEGs, the agriGO web-based program (http://systemsbiology.cau.edu.cn/agriGOv2/#) [37] was used to perform GO enrichment analysis of the DEGs. The KEGG (http://www.genome.jp/kegg/) database [98] was used to analyze the functional involvement of DEGs in various metabolic pathways. Additionally, the KOBAS 2.0 web server (http://kobas.cbi.pku.edu.cn/) [99] was used to test the statistical enrichment of DEGs in KEGG pathways [39]. Using hypergeometric test, based on the Student’s *t*-test, all KEGG pathways that had a *q* value < 0.01 were considered to be significantly enriched.

### 4.8. Quantitative Real Time-PCR (qRT-PCR) Analysis

To validate the assembled sequences and the expression profiles obtained by illumina RNA-seq, quantitative real-time PCR (qRT-PCR) was performed. Twenty DEGs were randomly selected and gene-specific primers were designed for qRT-PCR using Primer Premier 5 Designer (Premier Biosoft International, Palo Alto, CA, USA). Total RNA was isolated from seedling leaves as already described above in Section 4.3. Independent RNA from YE8112 and MO17 inbred lines leaf samples, and from control and drought-stress conditions was prepared for qRT-PCR analysis. HiFiscript cDNA Synthesis Kit (CWBIO, Beijing, China) was used as per the manufacturer’s protocol to perform cDNA synthesis, with 1 µg of total RNA being reverse-transcribed in 25 µL (total volume) reaction. The qRT-PCR analysis was carried out using Bestar^®^ SYBR Green qPCR Mastermix (DBI^®^ Bioscience, Ludwigshafen, Germany) in a Bio-Rad iQ5 thermo cycler (Bio-RAD, Hercules, CA, USA). The thermal profile for amplification was: 2 min at 95 °C; followed by 35 cycles, each consisting of 95 °C for 15 s, 55 °C for 30 s, and 72 °C for 15 s. The maize *GAPDH* (accession no. X07156) gene was employed as the endogenous control (forward primer: 5′-ACTGTGGATGTCTCGGTTGTTG-3′ and reverse primer: 5′-CCTCGGAAGCAGCCTTAATAGC-3′). Each PCR reaction (total volume of 20 µL) contained 10 µL of SYBR Green mix (TOYOBO, Osaka, Japan), 0.5 µL of each primer (50 pmol), and 2 µL of template cDNA; with all reactions being performed in triplicate. We monitored primer specificity and stability by dissociation curve analysis and agarose gel electrophoresis on a 3% agarose gel. Livak and Schmittgen’s [100] 2^−ΔΔ*C*t^ method was used to calculate the relative mRNA abundance in samples. We used SPSS statistical package (version 19.0, SPSS Institute Ltd., Armonk, NY, USA) to perform Pearson correlation coefficient analysis of RNA-seq versus qRT-PCR data. 

### 4.9. Statistical Analysis of Physiological Data

We used the SPSS software package (version 19.0; SPSS Institute Ltd., Armonk, NY, USA) to conduct the statistical analysis of physiological data by Fisher’s protected least significant differences (PLSD) test. The level of significance was set at *p* ≤ 0.05.

## 5. Conclusions

In this study, we have comprehensively compared the leaf transcriptome and physiological responses of drought-tolerant YE8112 and drought-sensitive MO17 maize inbred line seedlings after a seven-day drought exposure period. Resultantly, YE8112 seedlings maintained comparatively higher leaf relative water and proline contents, alongside increased peroxidase activity, but decreased MDA content, than MO17 seedlings. Using an RNA-seq-based approach, we mined out four critical sets of drought responsive DEGs, including 80 exclusive to YE8112, five DEGs of YE8112 also regulated in SD_TD, and four overlapping DEGs between the two inbred lines, among others. In drought-stressed YE8112, the DEGs were predominantly associated with the nitrogen metabolism, carbon metabolism, and amino-acid biosynthesis pathways, whilst those in MO17 were enriched in the ribosome pathway. More crucially, drought-responsive genes in tolerant line YE8112 were mainly implicated in stress signal transduction; cellular redox homeostasis maintenance; MYB, NAC, WRKY, and PLATZ transcriptional factor regulated; carbohydrate synthesis and cell wall remodeling; and protein ubiquitination processes. Our results enhance further elucidation of the molecular networks mediating drought tolerance, as well as providing foundational basis for further targeted cloning and downstream analysis of the identified specific individual genes. 

## Figures and Tables

**Table 1:** Physiological responses of the two contrasting maize inbred lines to drought stress.

Physiological Characteristics	Treatment Exposure Period (days) ^1^	Sensitive Inbred Line MO17		Tolerant Inbred Line YE8112	
		Control	Stress	Control	Stress
Leaf relative water content (%)	1	92.23 ± 0.1763 b	90.33 ± 0.8819 c	94.63 ± 0.2603 a	92.53 ± 0.1202 b
	3	93.03 ± 0.0882 a	88.53 ± 0.1202 b	93.70 ± 0.4163 a	89.90 ± 0.3056 b
	5	92.83 ± 0.4910 a	70.43 ± 0.1202 c	94.60 ± 0.4042 a	86.53 ± 0.2848 b
	7	92.73 ± 0.2403 a	65.40 ± 0.2082 c	93.80 ± 0.3215 a	81.80 ± 1.0214 b
Proline content (µg·g^−1^ FW)	1	88.62 ± 0.3358 a	80.45 ± 0.6265 b	73.99 ± 0.7420 c	89.95 ± 0.3259 a
	3	88.78 ± 0.1981 c	93.68 ± 0.5860 b	74.29 ± 1.1873 d	97.19 ± 1.0451 a
	5	88.69 ± 0.5200 c	99.78 ± 1.3309 b	75.25 ± 0.6094 d	108.00 ± 0.9824 a
	7	87.67 ± 1.2440 c	109.21 ± 0.7280 b	78.07 ± 0.9696 d	121.96 ± 0.7967 a
Peroxidase activity (unit·mg^−1^ protein FW min^−1^)	1	0.2653 ± 0.0028 b	0.2747 ± 0.0023 b	0.2590 ± 0.0012 c	0.3083 ± 0.0035 a
	3	0.2630 ± 0.0059 c	0.3147 ± 0.0015 b	0.2557 ± 0.0008 c	0.3553 ± 0.0047 a
	5	0.2530 ± 0.0064 d	0.3677 ± 0.0020 b	0.2643 ± 0.0052 c	0.3847 ± 0.1186 a
	7	0.2563 ± 0.0043 c	0.4033 ± 0.0039 b	0.2653 ± 0.0073 c	0.4350 ± 0.0012 a
MDA content (µmol·g^−1^ FW)	1	0.0119 ± 0.00003 b	0.0128 ± 0.00024 a	0.0118 ± 0.00067 b	0.0119 ± 0.00006 b
	3	0.0117 ± 0.00031 c	0.0152 ± 0.00015 a	0.0117 ± 0.00018 c	0.0123 ± 0.00028 b
	5	0.0115 ± 0.00012 a	0.0207 ±0.0002 8 c	0.0119 ± 0.00023 a	0.0153 ± 0.00057 b
	7	0.0120 ± 0.00012 a	0.0220 ± 0.00005 c	0.0118 ± 0.00018 a	0.0204 ± 0.00036 b
Drought stress injury symptoms	At 7 days	Not visible	Leaves distinctly shriveled up	Not visible	Leaves remain green and relatively intact

^1^ Physiological indices were recorded at different time points, viz. 1, 3, 5, and 7 days after drought treatment exposure; FW, fresh weight. Data are presented as mean of three replicates ± standard deviation. Means with similar letter(s) within the same row and for a particular characteristic do not differ significantly at 5% level using least significant difference (LSD) test.

**Table 2:** Summary of RNA sequencing results for the twelve maize seedling leaf samples.

Sample ^1^	Rep ^2^	Total Reads ^3^	Clean Reads ^4^	GC Content (%) ^5^	% ≥ Q30 ^6^	Mapped Reads (%) ^7^	Uniq. Map Reads (%) ^8^	Multiple Map Reads (%) ^9^
YE8112CK	1	65,863,880	32,931,940	57.05	89.78	50,491,140 (76.66%)	48,782,990 (74.07%)	1,708,053 (2.59%)
YE8112CK	2	64,181,688	32,090,844	56.15	89.07	49,434,118 (77.02%)	47,831,879 (74.53%)	1,708,053 (2.59%)
YE8112CK	3	59,227,506	29,613,753	56.82	89.96	45,756,005 (77.25%)	44,156,338 (74.55%)	1,599,667 (2.70%)
YE8112D	1	57,696,300	28,848,150	56.47	90.17	45,284,984 (78.49%)	41,401,607 (77.82%)	1,466,413 (2.54%)
YE8112D	2	59,181,570	29,590,785	56.13	89.54	45,357,186 (76.67%)	43,736,983 (73.90%)	1,638,203 (2.77%)
YE8112D	3	53,205,054	26,602,527	56.53	90.16	41,401,607 (77.82%)	40,072,870 (75.32%)	1,152,150 (2.47%)
MO17CK	1	46,584,386	23,292,193	56.04	89.17	30,323,524 (65.09%)	29,171,374 (62.62%)	1,590,356 (3.34%)
MO17CK	2	47,641,294	23,820,647	55.87	88.99	31,266,355 (65.63%)	29,675,999 (62.29%)	1,590,356 (3.34%)
MO17CK	3	47,180,622	23,590,311	56.54	88.70	31,791,159 (67.38%)	27,907,153 (59.15%)	3,884,006 (8.23%)
MO17D	1	44,255,824	22,127,912	56.10	86.79	26,090,436 (58.95%)	24,995,448 (56.48%)	1,094,998 (2.47%)
MO17D	2	68,595,584	34,297,774	56.22	87.01	41,355,964 (60.29%)	38,616,715 (56.30%)	2,739,249 (3.99%)
MO17D	3	47,342,680	23,671,340	56.38	85.02	26,906,481 (56.83%)	24,564,920 (51.82%)	2,371,561 (5.01%)

^1^ Sample, treatment sample; YE8112CK, tolerant line YE8112 under water-sufficient (control) conditions; YE8112D, tolerant line under drought stress; MO17CK, sensitive line MO17 under control; MO17D, sensitive line under drought stress; ^2^ Rep., treatment repeat; ^3^ Total reads, total clean reads counted by single end; ^4^ Clean reads, total number of pair-end reads in clean data; ^5^ GC content, clean data GC content, that is, the percentage of G and C bases out of total bases in the data; ^6^ % ≥ Q30, the percentage of the bases with a data mass value greater than or equal to 30; ^7^ Mapped reads, the number of reads in the reference genome and the percentage in clean reads; ^8^ Uniq. map reads, the number of reads compared in one location to the reference genome and the percentage in the clean reads; ^9^ Multiple map reads, the number of reads compared in multiple locations to the reference genome and the percentage in clean reads.

**Table 3:** Expression patterns of the differentially expressed genes (DEGs) by group.

Comparison/Group ^1^	DEG Number ^2^	Up-Regulated ^3^	Down-Regulated ^4^
SC__TC	4331	1964	2367
SC__SD	754	329	425
TC__TD	129	49	80
SD__TD	5398	2485	2913

^1^ Comparison/group, experimental comparison group; SD, sensitive inbred line (MO17) under drought treatment conditions; SC, sensitive inbred line under well-watered (control) conditions; TD, tolerant inbred line (YE8112) under drought conditions; TC, tolerant inbred line under control conditions; an underscore between two line-treatment combinations implies comparison of those combinations; ^2^ DEG number, total number of differentially expressed genes (DEGs) in the group; ^3^ Up-regulated, number of DEGs whose expression levels were increased; ^4^ Down-regulated, number of DEGs whose expression levels were decreased by the treatment.

**Table 4:** Drought responsive differentially expressed genes (DEGs) specific to tolerant line YE8112.

Gene ID ^1^	Gene Name/Description ^2^	log2 FC ^3^	Expr. ^4^	FDR ^5^	*p*-Value ^6^	KEGG Pathway ^7^
*Zm00001d027242*	Granule-bound starch synthase 1	4.3867007	Up	6.57 × 10^−6^	0.0001190	--
*Zm00001d044136*	Glycerol-3-phosphate acyltransferase 1	3.814405	Up	1.09 × 10^−5^	0.0020520	Glycerophospholipid metabolism
*Zm00001d029906*	*BETA_EXPANSIN7*	2.8909994	Up	0.002383	0.0015080	--
*Zm00001d036676*	Putative B-box type zinc finger family protein	2.666452	Up	0.0034115	3.60 × 10^−9^	--
*Zm00001d011473*	*POTASSIUM_CHANNEL5*	2.5990283	Up	0.0020524	0.0000007	--
*Zm00001d013261*	Cysteine protease 1	1.9554585	Up	2.60 × 10^−8^	1.72 × 10^−9^	--
*Zm00001d038199*	Mildew resistance locus O (MLO)-like protein 1	1.9445226	Up	3.07 × 10^−6^	0.0135900	--
*Zm00001d044765*	Benzoate carboxyl methyltransferase	1.7383231	Up	0.0004904	0.0148800	--
*Zm00001d012220*	Putative ENTH/ANTH/VHS superfamily protein	1.7341537	Up	0.0008504	0.0000584	--
*2626*	N/A	1.7277288	Up	0.0039107	0.0061660	--
*Zm00001d044285*	*CALCINEURIN_B-LIKE10*	1.6791443	Up	7.19 × 10^−6^	0.0042720	--
*1650*	N/A	1.6197127	Up	0.0016858	0.0000231	--
*Zm00001d033985*	N/A	1.6129169	Up	0.0040434	0.0000005	--
*Zm00001d046998*	*LIGHT-DEPENDENT SHORT HYPOCOTYLS 5*	1.5404762	Up	0.0025583	0.0011140	--
*2197*	N/A	1.5333337	Up	7.19 × 10^−6^	0.0022920	--
*Zm00001d028399*	Thaumatin-like protein 1	1.532225	Up	4.36 × 10^−9^	2.63 × 10^−8^	--
*Zm00001d024268*	*NAC-TRANSCRIPTION_FACTOR_110*	1.5062543	Up	1.30 × 10^−6^	0.0001437	--
*Zm00001d011297*	*MYB-RELATED-TRANSCRIPTION_FACTOR_35*	1.4840701	Up	0.0037662	4.04 × 10^−9^	--
*Zm00001d024546*	*LATE_HYPOCOTYL_ELONGATION_PROTEIN_ORTHOLOG1*	1.4762326	Up	1.03 × 10^−5^	0.0001785	Circadian rhythm—plant
*Zm00001d031349*	Serine--glyoxylate aminotransferase	1.415994	Up	8.99 × 10^−7^	0.0003943	Alanine; glutamate; and carbohydrate metabolisms
*Zm00001d048622*	N/A	1.4131758	Up	1.02 × 10^−6^	0.0000001	--
*3628*	N/A	1.3978699	Up	0.0025583	0.0081300	--
*Zm00001d038049*	Putative *O*-glycosyl hydrolase	1.2684934	Up	0.00603	0.0459900	--
*Zm00001d017374*	Protein kinase superfamily protein	1.2481588	Up	0.0062081	0.0042520	--
*Zm00001d008222*	N/A	1.1376143	Up	0.0026542	0.0001437	--
*1682*	N/A	1.1285633	Up	0.0051255	1.04 × 10^−10^	--
*Zm00001d008808*	*MYB-RELATED-TRANSCRIPTION_FACTOR_24*	1.1148561	Up	0.0069804	0.0000014	--
*Zm00001d040639*	RING-H2 finger protein ATL3F	1.0980329	Up	0.0039389	0.0089120	--
*Zm00001d017918*	*TRICHOME BIREFRINGENCE-LIKE 20*	1.0976007	Up	0.0006213	1.40 × 10^−8^	--
*Zm00001d029154*	Inactive beta-amylase 9	1.0845327	Up	0.0034368	0.0020340	--
*Zm00001d003850*	Probable botrytis susceptible 1 interactor (BOI) -related E3 ubiquitin-protein ligase 2	1.0379841	Up	0.0008516	0.0001594	--
*Zm00001d046318*	Putative flavin adenine dinucleotide (FAD)-binding berberine family protein	−1.0302981	Down	0.0034368	0.0001458	--
*Zm00001d005118*	Sec14p-like phosphatidylinositol transfer family protein	−1.0555703	Down	0.0004904	0.0000696	--
*Zm00001d011428*	Urophorphyrin methylase 1	−1.0643172	Down	0.0051255	0.0002838	--
*Zm00001d044228*	THIAMINE_BIOSYNTHESIS2	−1.065855	Down	0.0097628	0.0002834	Thiamine metabolism
*Zm00001d015700*	Putative chloride channel-like protein CLC-g	−1.0848056	Down	0.0003217	0.0000007	--
*Zm00001d015504*	Protein phosphatase 2C isoform gamma	−1.0960379	Down	0.0051889	0.0018030	--
*Zm00001d034345*	*FERREDOXIN_NADP_REDUCTASE1*	−1.11374	Down	9.83 × 10^−5^	0.0134500	Photosynthesis
*Zm00001d028164*	Sulfate transporter 2.2	−1.1852218	Down	0.0008516	0.0002960	--
*Zm00001d038186*	Protein NRT1/PTR FAMILY 3.1	−1.2124124	Down	0.0001231	0.0216600	--
*Zm00001d019312*	*BETA_GLUCOSIDASE_AGGREGATING_FACTOR1*	−1.2524453	Down	0.0014524	0.0004082	--
*Zm00001d013202*	*G2-LIKE-TRANSCRIPTION_FACTOR_8*	−1.2938652	Down	0.0036596	0.0020330	--
*Zm00001d011648*	Nuclear pore complex protein NUP50A	−1.2960047	Down	0.0022073	0.0222900	--
*Zm00001d002126*	loricrin-related	−1.3017332	Down	0.0041743	0.0013570	--
*Zm00001d015025*	Adenosine monophosphate (AMP) binding protein	−1.3030892	Down	4.79 × 10^−6^	0.0002818	--
*Zm00001d049831*	Nodulin-like protein	−1.3069293	Down	1.24 × 10^−5^	7.30 × 10^−9^	--
*Zm00001d003116*	Major facilitator superfamily protein	−1.3071872	Down	0.0023919	0.0071570	--
*Zm00001d002069*	DNA topoisomerase 2	−1.3224108	Down	0.0014831	0.0024900	--
*Zm00001d023592*	Amino acid permease 2	−1.4219628	Down	0.0014831	0.0240500	--
*Zm00001d003457*	Plant AT-rich and zinc (PLATZ) domain-containing protein 3	−1.4228022	Down	0.0029883	0.0151800	--
*Zm00001d018738*	*PATHOGENESIS_RELATED_PROTEIN4*	−1.5048829	Down	0.0003217	0.0010230	Plant hormone signal transduction; plant–pathogen interaction
*Zm00001d007517*	Subtilisin-like serine endopeptidase family protein	−1.5493362	Down	0.0011401	0.0005202	--
*Zm00001d021653*	Glucose-6-phosphate/phosphate translocator 2	−1.5573945	Down	0.0040043	4.00 × 10^−7^	--
*Zm00001d017279*	*PHENYLALANINE_AMMONIA_LYASE7*	−1.7207427	Down	0.0070058	2.595 × 10^−7^	Phenylalanine metabolism; phenylpropanoid biosynthesis

^1^ Gene ID, unique gene identifying number in the Maize Genetics and Genomics Database (Maize GDB); ^2^ Gene name/description, name or description of the gene identified by the given Gene ID; ^3^ Log2 FC, fold change, is expressed as the ratio of intensities of up-regulated or down-regulated genes between drought stress treatments and control (well-watered conditions); all the negative fold change values means that the genes were down-regulated. All the positive fold change values means the genes were up-regulated; ^4^ Expr., gene relative expression, with up meaning up-regulation and down meaning down-regulation; ^5^ FDR, false discovery rate, that is, the corrected *p*-value, set at <0.001 as the level at which gene differential expression was accepted as significant; ^6^ *p*-value, statistical level (using Student’s *t* test) below < 0.05; ^7^ KEGG pathways, Kyoto Encyclopedia of Genes and Genomes (KEGG), metabolic pathways in which the identified gene was found to be significantly enriched; N/A, means the gene is a newly discovered gene, without any functional annotation ascribed to it at the present.

**Table 5:** Drought responsive DEGs of the tolerant line that were also differentially expressed between the tolerant and sensitive lines after drought treatment.

Gene ID	Gene Name/Description	Expression in TC_TD				Expression in SD_TD				KEGG Pathway
		Expr.	Log2FC	*p*-Value	FDR	Expr.	Log2FC	*p*-Value	FDR	
*Zm00001d051511*	*PLATZ-TF7*	Up	1.03869	1.287 × 10^−9^	0.00747	Down	−1.73755	1.03 × 10^−4^	8.48 × 10^−12^	_
*Zm00001d052139*	Nitrate reductase [NAD(P)H]	Down	−1.64472	5.367 × 10^−8^	0.000867	Down	−7.52945	1.95 × 10^−32^	1.92 × 10^−9^	Nitrogen metabolism
*Zm00001d052164*	Ferredoxin—nitrite reductase	Down	−1.0642	0.000162	0.003449	Up	4.192623	0.000165	0.007743	Nitrogen metabolism
*Zm00001d052247*	Shikimate kinase 1 chloroplastic	Down	−1.18798	2.60 × 10^−6^	0.009651	Down	−7.09198	2.37 × 10^−11^	2.00 × 10^−23^	Phenylalanine, biosynthesis; biosynthesis of amino acids
*Zm00001d053568*	N/A	Up	1.754627	1.83 × 10^−7^	2.6 × 10^−6^	Down	−1.75125	1.74 × 10^−3^	7.69 × 10^−7^	_

For Table columns description, please refer to Table 4 caption above.

**Table 6:** Common DEGs between lines (YE8112 and MO17) after drought treatment.

Gene ID	Gene Name/Description	Expression in TC_TD				Expression in SC_SD				KEGG Pathway
		log2 FC	Expres.	FDR	*p*-Value	log2 FC	Expres.	FDR	*p*-Value	
*Zm00001d007012*	Ribonucleoprotein A	−1.10345	Down	0.003932	3.979 × 10^−7^	−1.95005	Down	2.79 × 10^−6^	0.000617	--
*Zm00001d014863*	*MYB- RELATED TF 96*	1.121959	Up	8.85 × 10^−5^	0.002432	−1.19141	Down	0.001988	3.68 × 10^−6^	--
*Zm00001d026501*	*GLUTAMINE_SYNTHETASE1*	−1.92817	Down	3.62 × 10^−6^	0.00001242	−2.62577	Down	9.96 × 10^−19^	0.000174	--
*Zm00001d045919*	Pyrophosphate—fructose-6-phosphate 1-phosphotransferase subunit alpha 2	1.510247	Up	0.004543	0.00001574	−2.95099	Down	0.00041	1.118 × 10^−9^	Fructose and mannose metabolism

For Table columns description, please refer to Table 4 caption above.

## Supplementary Materials

Supplementary materials can be found at https://www.mdpi.com/1422-0067/20/6/1268/s1.

## Abbreviations

ABA	Abscisic acid
ABF/AREB	ABA-responsive element binding factor/ABA-responsive element binding
Ca^2+^	Calcium signals receptors/messengers
CBL	Calcineurin B-like
CDPKs	Calcium dependent protein kinases
CIPKs	CBL-interacting protein kinases
DEGs	Differentially expressed genes
DREB/CBF	Dehydration responsive element binding/C-repeat binding factor
ENTH/ANTH/VHS	Epsin N-terminal homology/AP180 N-terminal homology/Vps27, Hrs and STAM
GO	Gene ontology
HSPs	Heat shock proteins
K^+^	Potassium channels signal receptors
KEGG	Kyoto Encyclopedia of Genes and Genomes
LEA	Late embryogenesis abundant proteins
LHY	Late hypocotyl elongation protein
LSH5	Light dependent short hypocotyls 5
MAPK	Mitogen-activated protein kinases
MDA	Malondialdehyde
MYB	Myeloblastosis oncogene
NAC	**NAM**, **ATAF**_1_/_2_, and **CUC**_2_ domain proteins
*PFP alpha 1*	Pyrophosphate fructose-6-phosphate 1-phosphotransferase subunit alpha 1
PLATZ	Plant AT-rich sequence and zinc binding protein
POD	Peroxidases
qRT-PCR	Quantitative real-time polymerase chain reaction
RNA-seq	RNA sequencing
ROS	Reactive oxygen species
SnRK2	SNF1-related kinase 2
SOD	Superoxide dismutase
TBL20	Trichome birefringence-like 20
TF	Transcription factor
TR	Thioredoxin reductase
WRKY	TF family denoted by protein domain composed of a conserved WRKYGQK motif and a zinc-finger domain
