# Supplementary material for: Key Maize Drought-Responsive Genes and Pathways Revealed by Comparative Transcriptome and Physiological Analyses of Contrasting Inbred Lines
Source: Int J Mol Sci. 2019 Mar 13;20(6):1268. doi: 10.3390/ijms20061268 (PMC6470692; doi:10.3390/ijms20061268)
Supplement: Supplementary file 1 [file ijms-20-01268-s001.zip › ijms-448766-SI-final/Supplementary Figures.pdf]

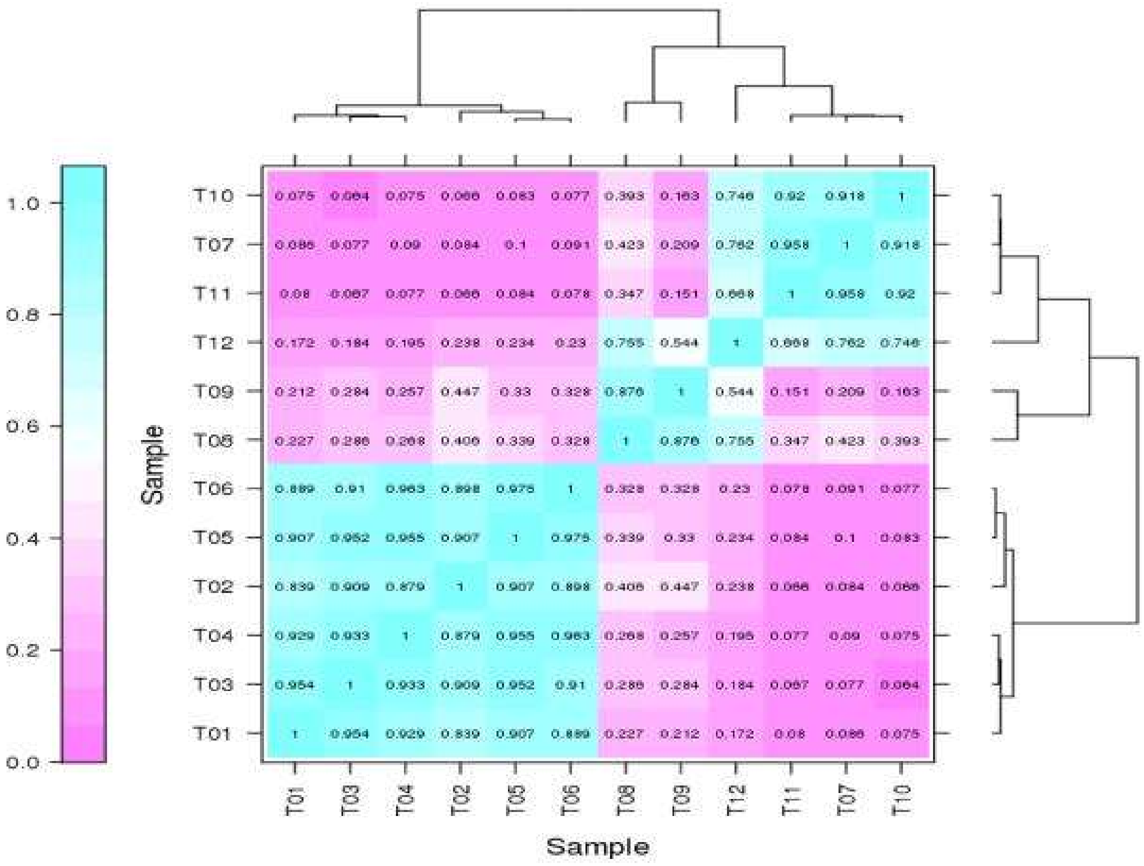

Figure S1. Pearson correlation coefficient analysis of biological replicates of different samples within two maize inbred lines with contrasting drought tolerance under different treatment conditions. The correlation coefficient (R^2) between two corresponding (x- and y-axis) samples was calculated based on the FPKM (fragments per kilobase of exon model per million mapped reads) values of those samples. The x- and y-axis shows the corresponding biological samples for different treatment conditions. T01-T03, tolerant line YE8112 under water-sufficient (control) condition; T04-T06, tolerant line YE8112 under water-deficit (drought) condition; T07-T09, sensitive line MO17 under control condition; T10-T12, sensitive line MO17 under drought condition.

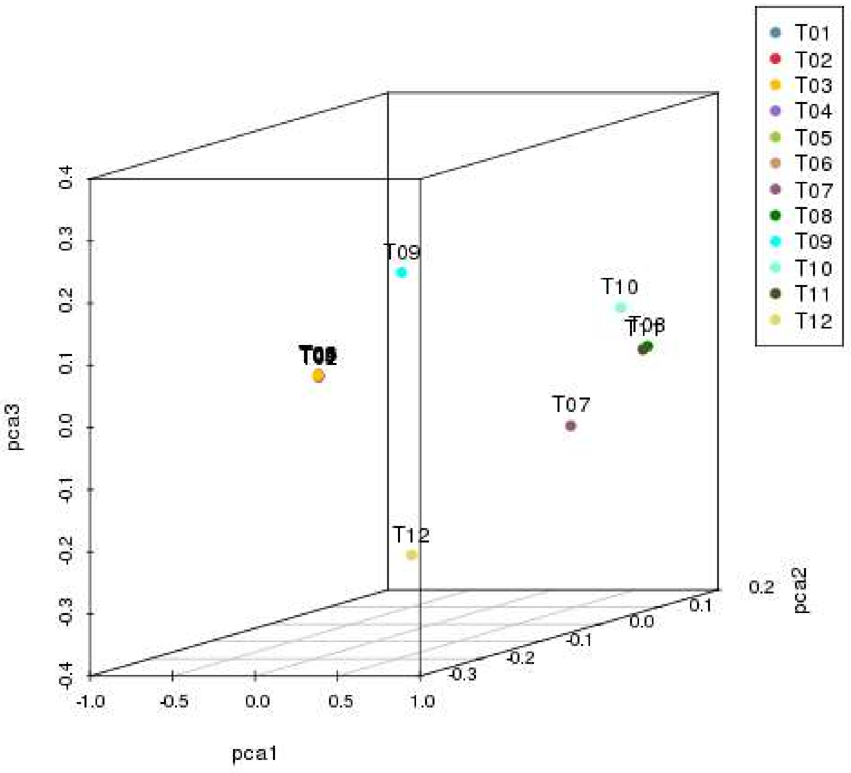

Figure S2. Principal Component Analysis (PCA) of the similarities and differences between the twelve samples used for RNA-Sequencing. The PCA analysis showed that there was low consistence amongst the three replications of the two (one drought-stress and one control) samples probably due to a technical failure. Thus, sequencing results were analyzed by retrieving samples T07 (MO17 water control) and T12 (MO17 water deficit).

Supplementary Figure 3 (Top 20 KEGG Pathways in Different Experimental Comparisons)

a) **Pathways enriched in TC TD comparion**

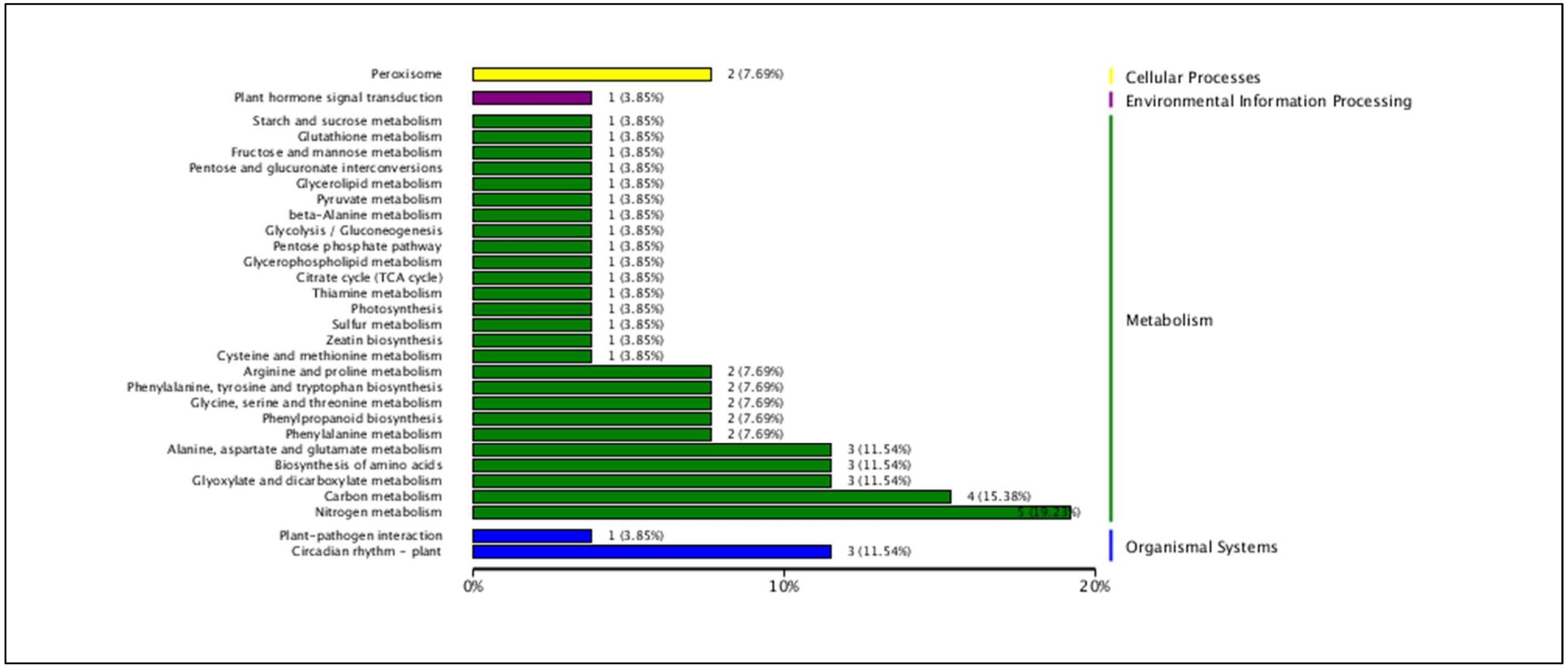

b) <u>Pathways enriched in SD_TD comparion</u>

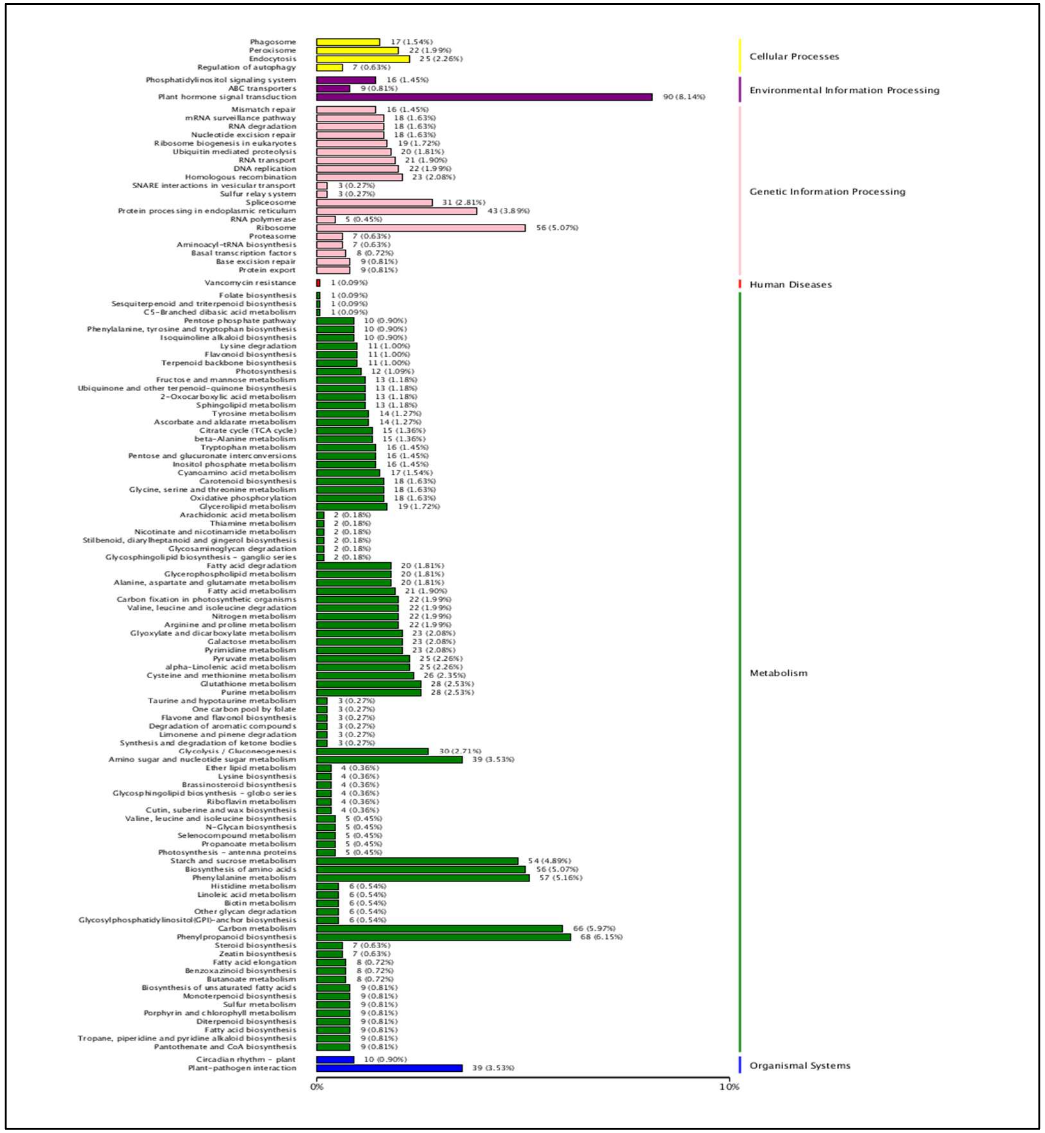

c) Pathways enriched in SC_SD comparion

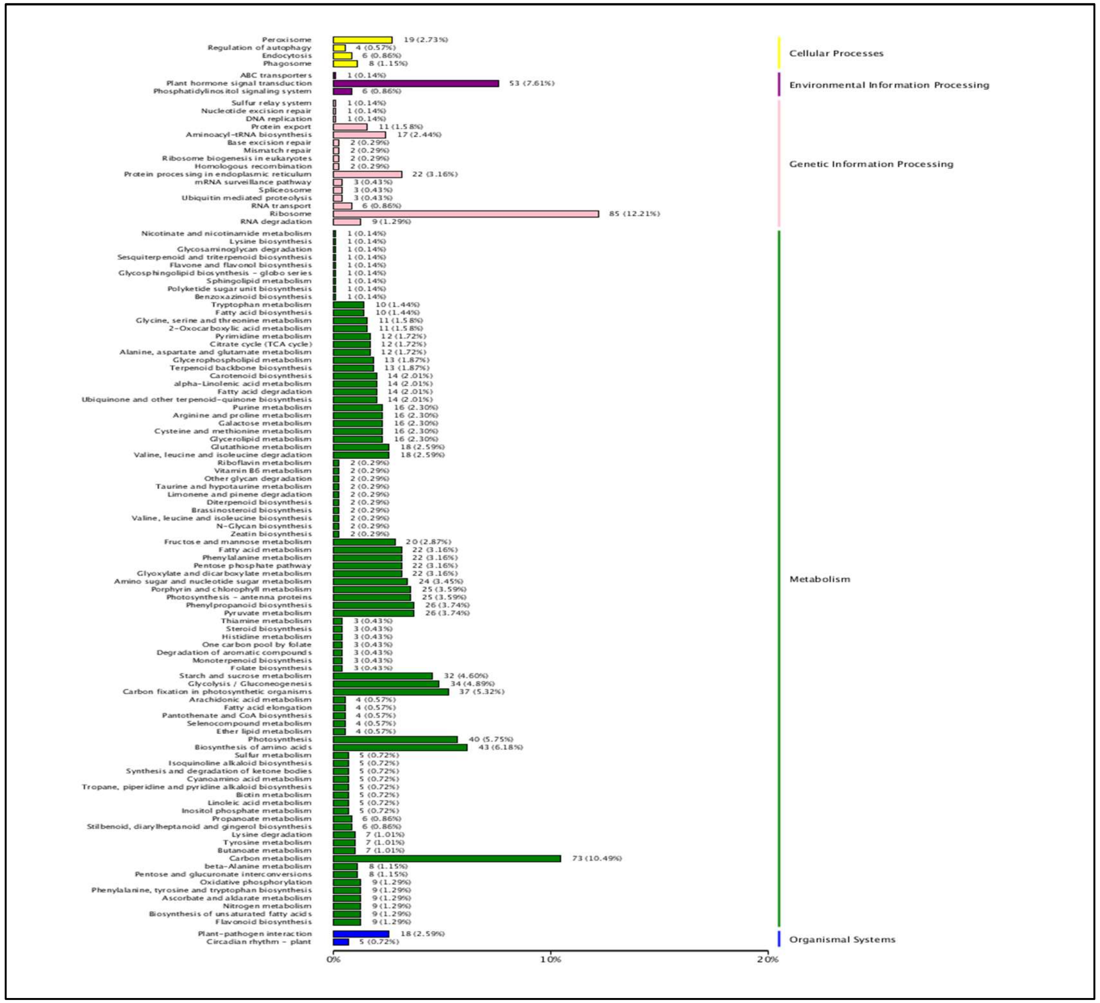

Figure S3. Top 20 KEGG pathway enrichment analysis of the differentially expressed genes (DEGs) in different experimental comparisons. (**A**) TC_TD comparison; (**B**) SD_TD comparison; and, (**C**) SC_SD comparison. The whole number above the bar (blue) graph represents number of DEGs enriched in the corresponding pathway.

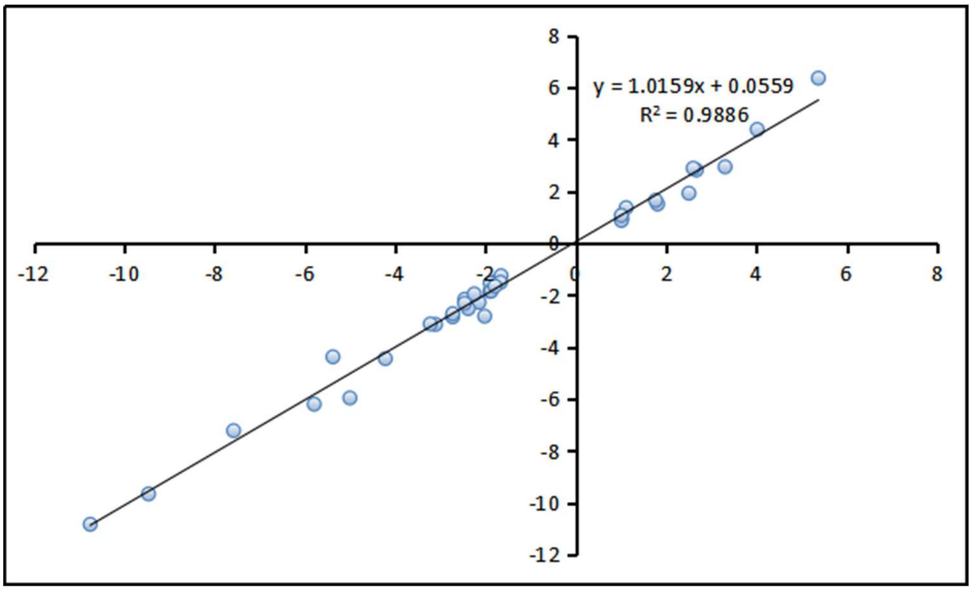

Figure S4. Validation of RNA-seq expression data by qRT-PCR analysis. Validation was performed using 20 randomly selected differentially expressed genes (DEGs). The plots demonstrate the expression ratio in log scale with base of two. The X-axis indicates qRT-PCR log scale; the Y-axis indicates RNA-seq log scale.

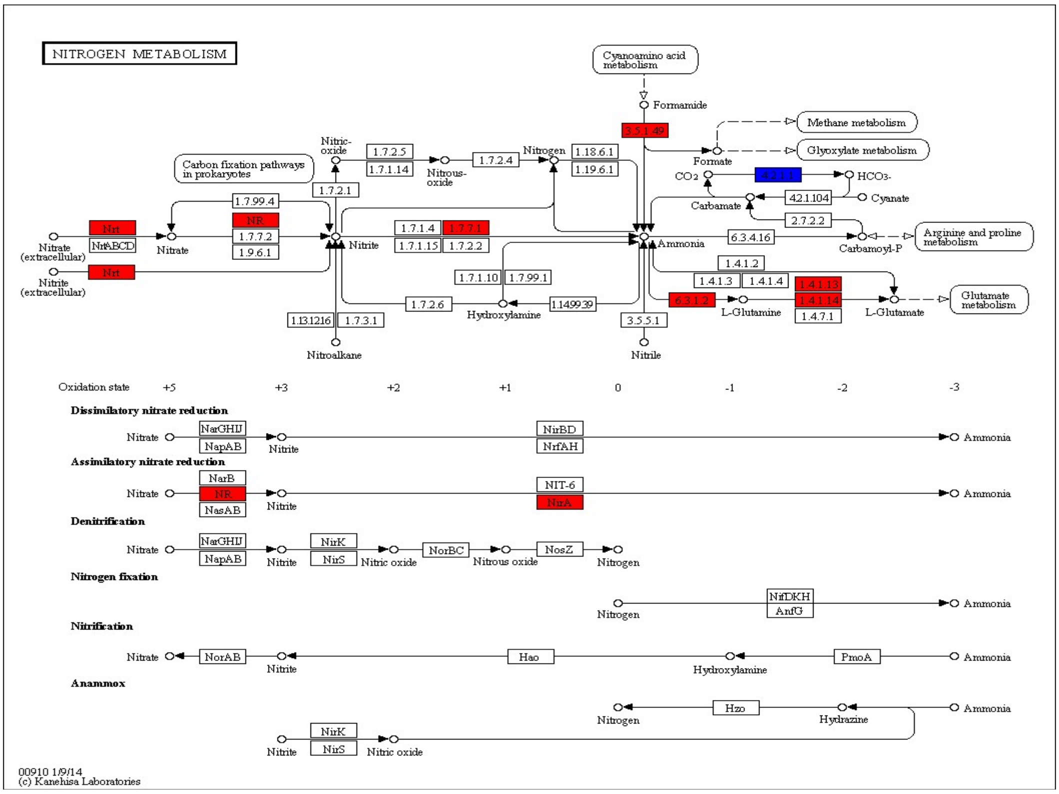

(**A**)

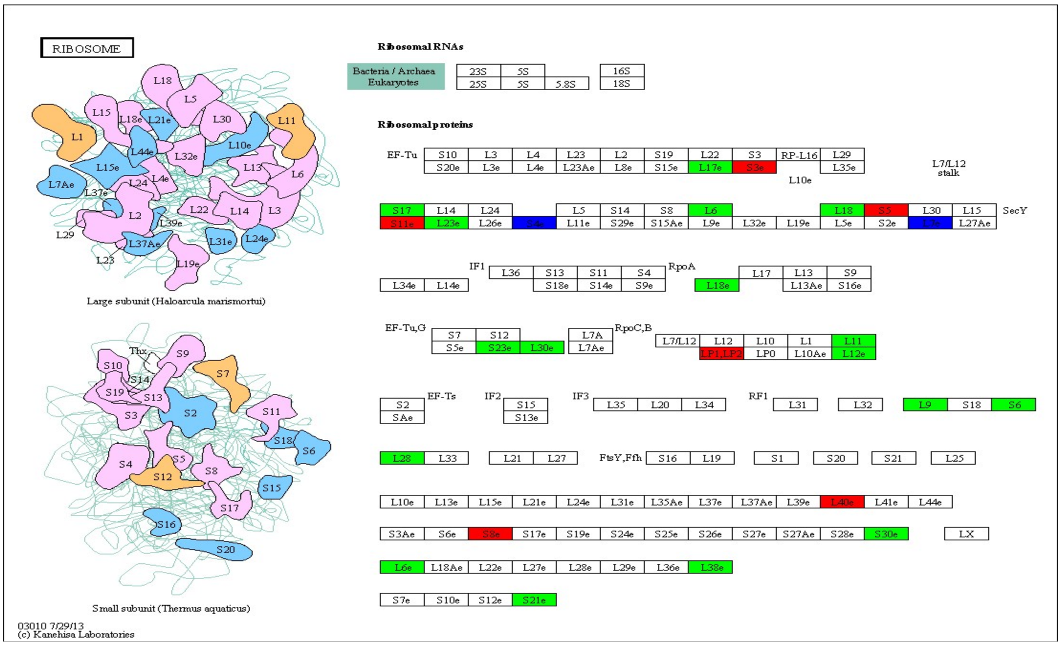

(**B**)

Figure S5. Most significantly enriched metabolic pathways in the two contrasting maize inbred lines under drought stress. (**A**) Nitrogen metabolism pathway significantly enriched in tolerant line YE8112; (**B**) Ribosome pathway significantly enriched in sensitive line MO17. Up-regulated genes are shown in red, down-regulated genes in green, and genes expressed in both cases are shown in blue.
