# Supplementary material for: Key Maize Drought-Responsive Genes and Pathways Revealed by Comparative Transcriptome and Physiological Analyses of Contrasting Inbred Lines
Source: Int J Mol Sci. 2019 Mar 13;20(6):1268. doi: 10.3390/ijms20061268 (PMC6470692; doi:10.3390/ijms20061268)
Supplement: Supplementary file 1 [file ijms-20-01268-s001.zip › ijms-448766-SI-final/Supplementary Tables.zip!/Supplementary Tables/Supplementary Table 8.docx]

International Journal of Molecular Sciences

Key maize drought-responsive genes and pathways revealed by comparative transcriptome and physiological analyses of two contrasting inbred lines

Tinashe Zenda ^1,2^ ^†^, Songtao Liu ^1,2 †^, Xuan Wang ^1,2^, Guo Liu ^1,2^, Hongyu Jin ^1,2^, Anyi Dong ^1,2^, Yatong Yang ^1,2^ and Huijun Duan^1, 2,^*

^1^	Department of Crop Genetics and Breeding, College of Agronomy, Hebei Agricultural University, Baoding 071001, China

^2^	North China Key Laboratory for Crop Germplasm Resources of the Education Ministry, Hebei Agricultural University, Baoding 071001, China

*****	Corresponding Author: hjduan@hebau.edu.cn; Tel.: +86-1393-1279-716

**^†^**	These authors contributed equally to this work

**Supplementary Table 11.** Summary of the functional annotation of assembled (differentially expressed genes) DEGs

| Annotated Database ^1^ | New gene number ^2^ | TC_TD ^3^ | SC_SD ^4^ | TC_SC ^5^ | TD_SD ^6^ |
| --- | --- | --- | --- | --- | --- |
| COG | 131 | 48 | 293 | 1342 | 1702 |
| GO | 592 | 106 | 649 | 3097 | 3965 |
| KEGG | 167 | 42 | 263 | 1131 | 1486 |
| Swiss-Port | 390 | 98 | 570 | 2743 | 3476 |
| eggNOG | 900 | 124 | 736 | 3831 | 4853 |
| NR | 1602 | 126 | 746 | 4232 | 5287 |
| All | 1618 | 128 | 746 | 4240 | 5294 |

^1^ Annotated database, the database used for functional annotation; ^2^ New gene number, the number of novel genes predicted by the corresponding database`s annotation information; ^3^ TC_TD, ^4^ SC_SD: tolerant and sensitive lines before and after drought treatment, respectively; ^5^ TC_SC, ^6^ TD_SD: tolerant and sensitive lines under water sufficient condition and water shortage condition, respectively. The third column to the last represents the number of DEGs that are annotated by the functional database; COG, clusters of orthologous groups; GO, gene ontology; KEGG, the Kyoto encyclopedia of genes and genomics; Swiss-Prot, a manually annotated protein sequence database; eggNOG, evolutionary genealogy of genes: Non-supervised orthologous groups; NR, non-redundant protein sequence; All, the total of differentially expressed genes in the data set/comparison group.
